# E‐selectin affinity glycoproteomics reveals neuroendocrine proteins and the secretin receptor as a poor‐prognosis signature in colorectal cancer

**DOI:** 10.1002/1878-0261.13733

**Published:** 2024-11-07

**Authors:** Sofia Cotton, Dylan Ferreira, Marta Relvas‐Santos, Andreia Brandão, Luís Pedro Afonso, Andreia Miranda, Eduardo Ferreira, Beatriz Santos, Martina Gonçalves, Paula Lopes, Lúcio Lara Santos, André M. N. Silva, José Alexandre Ferreira

**Affiliations:** ^1^ Portuguese Oncology Institute of Porto (IPO‐Porto)/Porto Comprehensive Cancer Center (P.ccc) Raquel Seruca Portugal; ^2^ ICBAS ‐ School of Medicine and Biomedical Sciences University of Porto Portugal; ^3^ i3S – Instituto de Investigação e Inovação em Saúde Universidade do Porto Portugal; ^4^ LAQV‐REQUIMTE, Department of Chemistry and Biochemistry, Faculty of Sciences University of Porto Portugal; ^5^ Pathology Department Portuguese Oncology Institute of Porto Portugal; ^6^ Health School of University Fernando Pessoa Porto Portugal; ^7^ Department of Surgical Oncology Portuguese Oncology Institute of Porto Portugal

**Keywords:** cancer glycoproteome, colorectal cancer, E‐selectin, metastasis, secretin receptor

## Abstract

Colorectal cancer (CRC) cells express sialylated Lewis antigens (sLe), crucial for metastasis via E‐selectin binding. However, these glycoepitopes lack cancer specificity, and E‐selectin‐targeted glycoproteins remain largely unknown. Here, we established a framework for identifying metastasis‐linked glycoproteoforms. More than 70% of CRC tumors exhibited overexpression of sLeA/X, yet without discernible associations with metastasis or survival. However, The Cancer Genome Atlas (TCGA) analysis unveiled differing expression patterns of sLeA/X‐related glycogenes correlating with disease severity, indicating context‐dependent regulation by distinct glycosyltransferases. Deeper exploration of metastatic tumor sialoglycoproteome identified nearly 600 glycoproteins, greatly expanding our understanding of the metastasis‐related glycoproteome. These glycoproteins were linked to cell adhesion, oncogenic pathways, and neuroendocrine functions. Using an in‐house algorithm, the secretin receptor (SCTR) emerged as a top‐ranked targetable glycoprotein. Tumor screening confirmed SCTR's association with poor prognosis and metastasis, with N‐glycosylation adding cancer specificity to this glycoprotein. Prognostic links were reinforced by TCGA‐based investigations. In summary, SCTR, a relatively unknown CRC glycoprotein, holds potential as a biomarker of poor prognosis and as an E‐selectin ligand, suggesting an unforeseen role in disease dissemination. Future investigations should focus on this glycoprotein's biological implications for clinical applications.

## Graphical abstract

Screening colorectal tumors for glycoproteins linked to disease progression has revealed several potential therapeutic targets. The secretin receptor (SCTR), identified as a marker of poor prognosis and metastasis in colorectal cancer, has been shown to be a key carrier of N‐glycans with sialylated Lewis antigens. These cancer‐specific glycoproteoforms provide a strong rationale for precise targeting in cancer therapy.

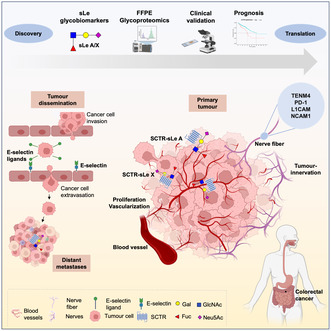

**Abbreviations**
BSA: bovine serum albumin
CA19‐9: carbohydrate antigen 19‐9
CD34: cluster of differention 34
CRC: colorectal cancer
DAPI: 4′,6‐diamidino‐2‐phenylindole
DTT: dithiothreitol
FFPE: formalin‐fixed paraffin‐embedded
FUT: fucosyltransferase
GDC: genomic data common
GlcNAc: *N*‐acetylglucosamine
GPCR: G‐protein coupled receptors
GuHCl: guanidine hydrochloride
HR: hazard ratio
IgG: immunoglobulin G
IL: interleukin
IP: immunoprecipitation
KOH: potassium hydroxide
M0: no distant metastases
M1: distant metastases
MAPK: mitogen‐activated protein kinase
MS: mass spectrometry
MUC: mucin
OS: overall survival
PFS: progression‐free survival
PLA: proximity ligation assay
PNGase F: peptide‐*N*‐glycosidase F
RT: room temperature
SCT: secretin
SCTR: secretin receptor
sLe: sialylated Lewis antigens
TCGA: The Cancer Genome Atlas
TNF: tumor necrosis factor

## 1 Introduction

Colorectal cancer (CRC) remains a significant health burden, ranking high in both incidence and mortality rates [1]. The challenges associated with CRC are amplified by late‐stage diagnoses and the limited efficacy of existing therapeutics against advanced disease [2]. To address these limitations, there is a crucial need to identify molecular signatures associated with metastasis for precise cancer targeting.

Recent progress in glycomics has significantly advanced our understanding about the alterations in the glycocalyx accompanying CRC development and progression [3, 4, 5]. Utilizing high‐throughput mass spectrometry, researchers have comprehensively characterized primary tumors, metastatic sites, and healthy colon mucosa from diverse patient populations [5, 6]. These investigations have unveiled key alterations, particularly the expression of (sialyl‐)LewisA/X antigens (sLeA/X) that cap extended *N*‐ as well as O‐glycosidic chains on glycoproteins [5]. Traditionally linked to poor prognosis, these antigens play a pivotal role in mediating metastasis by facilitating cancer cell adhesion to endothelial cells, intravasation into the bloodstream, and homing to distant sites [3]. This makes sLe antigens attractive targets for controlling metastasis. Glycomics analyses have identified a substantial presence of glycoproteins in CRC cases featuring core 2 O‐glycans with terminal sLe antigens, a characteristic not found in normal colon tissue [5]. This pattern is notably pronounced in mucinous adenocarcinomas (T2 and T3), commonly classified as MSI tumors [7]. However, it is worth noting that sLe antigens may also be present in core 3 structures in normal colon mucosa, albeit with a downregulation in cancer [5]. In summary, these findings provide a compelling molecular basis for targeting sLe glycoepitopes in CRC. However, they also emphasize the imperative for a comprehensive characterization of the sLe‐glycoproteome linked to metastasis, envisioning the essential cancer‐specificity required for the development of targeted therapeutics.

Building upon these findings, this study focuses on creating a framework to examine the CRC glycoproteome, aiming to identify molecular features linked to metastasis. This involves a combination of glycomics and E‐selectin affinity glycoproteomics to identify sLe‐glycoproteoforms in metastatic tumors. Additionally, we employed an in‐house developed algorithm that draws from a well‐established protein expression data repository to pinpoint unforeseen and potentially clinically relevant and cancer‐specific glycosignatures [8, 9, 10]. These glycosignatures have been further validated in clinical samples and their cancer specificity confirmed by screening of healthy tissues. We anticipated that this may constitute a relevant milestone towards a comprehensive understanding of metastases signatures, paving the way for future advancements in patient care.

## 2 Materials and methods

### 2.1 Patient sample set and healthy human tissues

SLeA, sLeX, and SCTR were retrospectively analyzed in 35 (FFPE) advanced CRC tissues obtained from the IPO‐Porto biobank, reflecting the incidence of the disease. The patient cohort included 15 female and 20 male patients, aged between 28 and 76 years (median age: 61 years), who underwent surgical resection of colorectal carcinomas at IPO‐Porto from 2005 to 2012. The expression of these markers was also evaluated in six lymph node, seven hepatic, and three peritoneal metastases, which are available for some of these primary tumors. The time of follow‐up was on average 45 months (±23 months). Overall survival (OS) was defined as the period between surgery and the last follow‐up evaluation on the subsequent years or the occurrence of death by cancer. All clinicopathological information used to assess the clinical relevance of SCTR, sLeA, and sLeX expressions is summarized in Table S1. Clinicopathological data were extracted from the patients' medical records. This study received approval from the IPO institutional Ethics Committee (Approval No. CES 64/2017, dated March 16, 2017) and was conducted following informed written consent from all participating patients. All study methodologies were performed according to the standards set by the declaration of Helsinki. Overall survival (OS) was calculated from the date of surgical intervention to the date of death attributable to cancer. Recurrence was defined as the manifestation of the disease, either locally or at a distant site, following the initial treatment. A series of healthy tissues recovered from autopsies, including appendix, colon, lung, liver, pancreas, stomach, skin, testis, and thyroid, were also screened for these sialoglycans and the SCTR to assess cancer specificity. Tissues from three different individuals were analyzed.

### 2.2 Immunohistochemistry

Three‐micrometer sections from FFPE colorectal tumor samples was screened for sLeA, sLeX, and SCTR expression. Briefly, tissue sections were deparaffinized, hydrated, and subjected to antigen retrieval using a low pH citrate buffer (Vector Laboratories, Newark, CA, USA). Subsequently, the sections were treated with a 3% hydrogen peroxide solution (Leica Biosystems, Deer Park, IL, USA) to inhibit endogenous peroxidase activity. To minimize nonspecific binding, the sections were exposed to Protein Block (Leica) before incubation with monoclonal antibody anti‐CA19.9 (1 : 100; ab116024; Abcam, Cambridge, UK), monoclonal antibody anti‐sLeX (1 : 100; 368102; BioLegend, San Diego, CA, USA) targeting dimeric sLex and sLex with long carbohydrate attachments, and polyclonal antibody anti‐Secretin Receptor (1 : 100; ab224236; Abcam) for the determination of sLeA, sLeX, and SCTR, respectively. Antibody binding to the tissue sections was assessed using the Novolink Max Polymer DS Kit (Leica), following the manufacturer's protocol. A double‐blinded analysis was carried out by independent observers and subsequently validated by an experienced pathologist. Immunohistochemical analysis included the assessment of expression patterns, encompassing extension (percentage of positive tumor cells) and intensity (degree of chromogenic deposit), as well as the subcellular location of the staining. Quality assurance measures involved parallel testing of both positive and negative controls. Enzymatic controls utilizing α‐neuraminidase from *Clostridium perfringens* (Sigma‐Aldrich, St. Louis, MO, USA) were employed to ensure the specificity and accuracy of the immunohistochemical results for the two sialoglycans (sLeA and sLeX). CRC tissues used in glycoproteomics studies were further screened for the vascular marker CD34 (1 : 100; M7165; Dako, Agilent; Santa Clara, CA, USA) and two neuroendocrine markers (Synaptophysin and Chromogranin A – 1 : 100, M7315, Dako; 1 : 1000, M0869, Dako, respectively).

### 2.3 Glycomics

N‐ and O‐glycomics were performed on the three metastatic colorectal tumors used for glycoproteomics characterization to disclose the nature of the glycans terminated with sLe epitopes, as previously described [11, 12]. Briefly, FFPE tissues were deparaffinized, rehydrated, and subjected to heat‐induced antigen retrieval using a citrate‐based solution (Vector Laboratories). Then, proteins were denatured and reduced by incubation with 150 μL denaturation mix (145 μL 8 m GuHCl and 5 μL 200 mm DTT) at 60 °C for 30 min. N‐glycan release was achieved after digestion with PNGase F (1 U/10 μg protein at 37 °C overnight; Promega, Madison, WI, USA). Released N‐glycans were hydrolysed with 25 μL of 100 mm ammonium acetate at pH 5 for 1 h at room temperature (RT), removing the glycosylamine form of the nonreducing terminus, and subsequently reduced with 20 μL of 1 m NaBH_4_ in 50 mm KOH for 3 h at 50 °C. The reaction was quenched by adding glacial acetic acid. O‐glycans were released by reductive β‐elimination (1 m NaBH_4_ in 50 mm KOH overnight at 50 °C). Finally, reduced N‐ and O‐glycans samples were desalted using a cation exchange resin (AG 50W‐X8; Bio‐Rad, Hercules, CA, USA). All glycans were permethylated and analyzed by reverse phase nanoLC‐ESI‐MS/MS as previously described by us [9], using a 3000 Ultimate nano‐LC coupled to a QExactive mass spectrometer (Thermo Fisher Scientific, Waltham, MA, USA). Glycan structures were identified considering previous knowledge on glycosylation, chromatography retention times, *m/z* identification, and corresponding product ion spectra. Manual annotation was supported by GlycoWorkbench tool [13, 14].

### 2.4 Glycoproteomics and data curation

Glycoproteome characterization was conducted as previously described [14, 15]. Briefly, proteins were extracted from three metastatic CRC FFPE tissues using the QProteome FFPE tissue kit (QIAGEN, Hilden, Germany) according to the manufacturer's instructions and pooled to a total of 700 μg of protein. Samples were then enriched for glycoproteins carrying sLe antigens by E‐selectin affinity chromatography, as previously described by our group [16], using a recombinant mouse E‐selectin Fc Ig chimera protein (575‐ES‐100; R&D Systems, Minneapolis, MN, USA). Negative controls included immunoprecipitations (IPs) with an IgG1 isotype control (LTI‐02‐6102; Thermo Fisher Scientific) and with the E‐selectin chimera in the absence of Ca^2+^. After electrophoresis, glycoproteins were reduced and digested with chymotrypsin, following the protocol described in Fernandes et al. [9]. The mass spectrometry analysis was performed by nanoLC‐MS/MS using an Ultimate 3000 RSLCnano system coupled to a QExactive mass spectrometer (Thermo Fisher Scientific). Eluent A was aqueous formic acid (0.2%), while eluent B was formic acid (0.2%) in acetonitrile. Briefly, samples were injected into a trapping column (C18 PepMap 100, 5 μm particle size) and washed with an isocratic flux of 98% eluent A and 2% eluent B at a flow rate of 10 μL·min^−1^. After 3 min, the flux was redirected to the analytical column (EASY‐Spray C18 PepMap, 100 Å, 150 mm × 75 μm I.D and 3 μm particle size) at a flow rate of 0.25 μL·min^−1^. The column temperature was set at 35 °C. Glycopeptide separation occurred using a linear gradient of 10–40% eluent B over 50 min. Column wash and re‐equilibration were warranted before the following injection. The mass spectrometer was operated in the positive ion mode, with an *m/z* range from 300 to 2000, with a spray voltage of 1.9 kV and a transfer capillary temperature of 275 °C. Q‐Exactive HF settings were full scan resolution 120 000, fragment scan resolution 15 000, fragment scan fixed first mass at 110 *m/z*, normalized collision energy 30%, isolation window 4.0 *m/z*. Mass spectrometry data were processed using the SequestHT search engine and the Percolator algorithm (Proteome Discoverer 3.0; Thermo Fisher Scientific) to validate protein identifications. Data were searched against the human membrane proteome from the SwissProt database. Chymotrypsin was selected as the digestion enzyme, considering up to two missed cleavage sites, a precursor ion mass tolerance of 10 p.p.m., and a product ion tolerance of 0.02 Da. Fixed and variable modifications included carbamidomethylcysteine (+57.021 Da) and oxidation of methionine (+15.995 Da), respectively. The following N‐glycans were also considered as variable modifications, building on glycome analysis: H5N4F1S1 (+2059.735 Da; asparagine); H5N4F1S2 (+2350.830 Da; asparagine). Alternatively, the following O‐glycans were considered: Hex(2)HexNAc(2)NeuAc(1)dHex(1) (+1167.418 Da), Hex(2)HexNAc(2)NeuAc(2)dHex(1) (+1458.513 Da), and Hex(3)HexNAc(3)NeuAc(2)dHex(2) (+1969.703 Da) on serine and threonine. The final glycopeptide list included protein species solely detected in E‐selectin pulldowns, identified with high confidence as well as with low confidence but presenting manually validated glycopeptides. Glycoproteins molecular and biological functions and subcellular localization were assessed by GO term analysis using cluego version 2.5.10 for cytoscape version 3.10.1 [17, 18, 19].

### 2.5 Target prioritization

The in‐house Target Score algorithm, building on the Human Protein Atlas database (proteinatlas.org) [20], was employed to prioritize glycoproteins for precise cancer targeting, as previously described by us [8].

### 2.6 *In situ* proximity ligation assay

SCTR‐sLeA/X glycoproteoforms were indirectly assessed in the three CRC tissues used for glycomics and glycoproteomics by proximity ligation assay (PLA) employing Duolink *in situ* Detection Reagents Red (Sigma‐Aldrich). After FFPE tissue deparaffinization and antigen retrieval with acid and heat treatment, the tissue sections were incubated overnight at 4 °C with primary antibodies: anti‐SCTR (ab224236; Abcam) and anti‐CA19‐9 (ab116024; Abcam) or anti‐sLeX (368102; BioLegend) in a humidity‐controlled chamber. The tissues were subsequently incubated with anti‐rabbit MINUS and anti‐mouse PLUS PLA probes at 37 °C for 1 h. Subsequent stages included a 30 min ligation process at 37 °C and a 120‐min amplification phase 37 °C to induce the formation of fluorescent rolling dots. Tissue sections were then stained with 4′,6‐diamidino‐2‐phenylindole (DAPI) for 10 min at RT and analyzed by fluorescence microscopy. The PLA results underwent evaluation by two independent observers and were further validated by a seasoned pathologist. Fluorescence images were acquired on a Leica DMI6000 FFW microscope using the las x software (Leica).

### 2.7 Immunoprecipitation and western blot

The SCTR was immunoprecipitated from a pool of proteins isolated from three FFPE tissues used for glycoproteomics on lysis buffer (50 mm Tris, 150 mm NaCl, 1% NP‐40, 0.5% sodium deoxycholate, 0.1% SDS) using the Pierce™ Direct IP Kit (Thermo Scientific). This involved a two‐stage approach: first, immunoprecipitating SCTR by antibody targeting, followed by enrichment of glycoproteoforms with affinity for E‐selectin. Briefly, the rabbit polyclonal SCTR (ab224236) used for immunohistochemistry and PLA was first immobilized in agarose beads according to the manufacturers' instructions. Afterwards, the beads were preblocked with 1% Bovine Serum Albumin (BSA; Sigma‐Aldrich) for 1 h at 4 °C and protein extracts were precleared using the BSA‐blocked agarose beads for 2 h at 4 °C to reduce unspecific binding. Then, the supernatant was incubated with the beads at 4 °C for 2 h, followed by overnight incubation with freshly BSA‐blocked agarose beads at 4 °C. After washing, the SCTR was eluted with 3% (v/v) acetic acid, dried under vacuum and resuspended in IP lysis buffer (25 mm Tris HCl pH 7.4, 150 mm NaCl, 1% NP‐40, 1 mm EDTA, 5% glycerol) with 2 mm CaCl_2_. The IP material was then incubated with a recombinant mouse E‐selectin Fc Ig chimera protein immobilized in agarose as previously described in Section 2.4. The IP products were resolved on a 4–20% gradient SDS/PAGE gel (Bio‐Rad) followed by protein blotting as described by Fernandes et al. [9], using anti‐SCTR mouse anti‐human SCTR monoclonal antibody (1 : 100, 1 h at RT; sc 166112; Santa Cruz Biotechnology, Dallas, TX, USA), anti‐CA19.9 (1 : 100, 1 h at RT; ab116024; Abcam) and anti‐sLeX (1 : 100; 368102; BioLegend). Chemiluminescence was captured and analyzed using a ChemiDoc XRS+ system with the image lab™ Software (Bio‐Rad).

### 2.8 Flow cytometry

Circulating leukocytes were screened for sialylated Lewis antigens and SCTR by flow cytometry. Peripheral blood samples were obtained from three female healthy donors at Immunohemotherapy Department of Centro Hospitalar São João (CHSJ), Porto, Portugal. Procedures were approved by the Hospital Ethics Committee (Protocol reference 260/11). Whole blood, after red blood cell lysis with RBC lysis buffer 1× (420301; BioLegend), was incubated with primary antibodies: rabbit anti‐sLeA (CA19‐9, NBP2‐53220; Novus Biologicals, Centennial, CO, USA), mouse anti‐sLeX (CD15s, 551344 BD), and anti‐secretin receptor (sc‐166112; Santa Cruz Biotechnology). Subsequently, leucocytes were incubated 30 min with anti‐rabbit Alexa 647 and anti‐mouse Alexa 488. Further incubation with anti‐CD45‐PE/Cy7 (368532; BioLegend) was performed. After additional washes, cells were acquired on a NAVIOS Flow Cytometer (Beckman Coulter, Brea, CA, USA). The FACS data were analyzed using the kaluza c software (version 1.2) (Beckam Coulter Life Sciences, Indianapolis, IN, USA) to discriminate cell populations, determine the mean fluorescence intensity (MFI) and the percentage of positive cells.

### 2.9 TCGA dataset analysis

Gene expression data of 623 colorectal (COADREAD dataset) tumor tissues and 51 normal adjacent tissues from the Genomic Data Common (GDC) database were downloaded from the UCSC Xena database (http://xena.ucsc.edu/). Additionally, clinicopathological characteristics, including age, gender, tumor stage, and histological type, as well as survival information, were also obtained. After excluding patients with missing clinical data, a final dataset comprising 598 tumor samples and 51 histologically normal adjacent tissue samples was included in the study. The clinical data for COADREAD patients are presented in Table S2. The prognosis value of glycosyltransferases involved in sialyl‐Lewis epitopes, SCTR and secretin (SCT) in CRC was performed by univariate, and subsequent multivariate Cox regression. Kaplan–Meier analysis and log‐rank test were used to compare OS and progression‐free survival (PFS) curves, using the “survival” and “survminer” r packages, respectively. Optimal cutoff value for each gene expression was determined using the surv_cutpoint function, for both OS and PFS outcomes. Shapiro–Wilk normality test was used to determine variable normality, and Wilcoxon rank test was performed to assess the difference between tumor stages and sample tissue types. Correlation analysis was performed using Spearman's correlation and visualized by correlograms using the “corrplot” r package. *P*‐value < 0.05 was considered as a statistically significant difference. All statistical analysis were performed using the r software (version 4.2.1) (R Foundation for Statistical Computing, Vienna, Austria).

### 2.10 Glycogenes on CRC patient samples

*ST3GAL3* gene expression was assessed by quantitative polymerase chain reaction (qPCR). Briefly, total RNA from FFPE tissues was isolated using High Pure FFPET RNA Isolation Kit (Roche, Basel, Switzerland), according to the manufacturer's instructions. RNA was converted to cDNA with the High‐Capacity cDNA Reverse Transcription Kit (Applied Biosystems, Thermo Fisher Scientific®, Waltham, MA, USA) according to the manufacturer's protocol. The mRNA levels were normalized to the expressions of glyceraldehyde‐3‐phosphate dehydrogenase (GAPDH), which were found to be the most stable reference genes under the studied conditions. The relative mRNA levels were calculated using the formula $2^{-\Delta \Delta C_{t}}$ as described by Livak and Schmittgen [21]. The efficiency of each probe was above 95% as determined by the manufacturer. All reactions were run in duplicates and experiments were performed in triplicates.

### 2.11 Statistical analysis

Mann–Whitney tests were performed to compare the expressions of sLeA, sLeX, and SCTR in nonmetastatic vs metastatic tumors from IPO‐Porto patients, after Shapiro–Wilk normality test. Chi‐squared tests were used to assess the statistical relationships between T stage, clinical stage and tumor sample type (primary tumor, lymph node metastasis and distant metastasis), sLe antigens, and SCTR expression (positive, negative). A significance threshold of 95% for the null hypothesis was applied.

## 3 Results

This work builds on a well‐established notion that sLe antigens are key mediators of metastasis by showing high affinity to E‐selectin. It highlights the abundant expression of these glycans in CRC and proposes a roadmap for identifying cancer‐specific glycoproteoforms, with implications for targeted therapeutics in the future. Moreover, the study aims to establish a foundation for identifying potentially clinically relevant molecular signatures for precision medicine, utilizing glycosylation as a starting point.

### 3.1 sLe antigens in CRC and healthy tissues

**Fig. 1:**
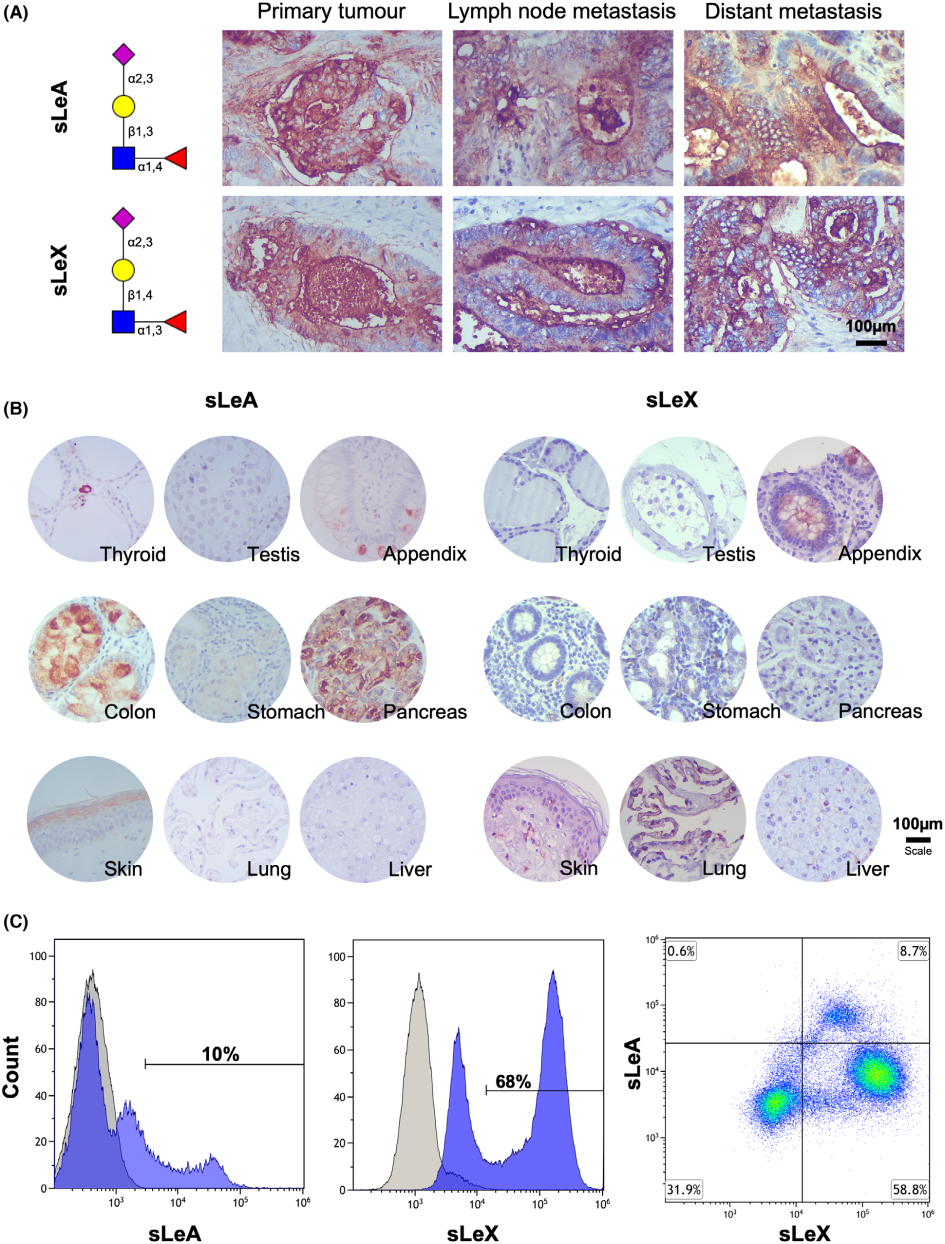
Sialylated Lewis antigens exhibit high expression levels in primary tumors, as well as in lymph node and distant metastases in contrast to healthy organs and leukocytes. (A) Sialyl Lewis A (sLeA) and sialyl Lewis X (sLeX) present a diffuse expression pattern in colorectal cancer (CRC) primary tumors and metastases. Over 75% of CRCs express sialylated Lewis antigens, regardless of their stage. Lymph node and distant metastases of tumors positive for sLe antigens also express these glycoepitopes, mirroring the pattern observed in the primary tumor. (B) Sialylated Lewis antigens present a restricted expression pattern in histologically normal human tissues. SLeA is mostly expressed in the colon and pancreas, whereas sLeX is mostly found in the lungs. Residuals expressions of these antigens could be found in the appendix and skin. (C) sLeX is significantly more expressed in circulating leukocytes in comparison to sLeA. Approximately 10% of circulating leukocytes express sLeA, whereas 68% express sLeX. Notably, sLeX is mostly expressed by granulocytes (76%), while approximately 90% of lymphocytes plus monocytes express both antigens.

We first devoted to characterizing the expression pattern of sLeA and sLeX in advanced CRC, building on a patient series of 35 tumors comprehending more advanced stages of the disease (stage III/IV). Over 75% of the tumors expressed both sLeA and sLeX antigens, in general in approximately 30% of the tumor area, in the same regions and without a defined expression pattern (Fig. 1A). Notably, the percentage of sLe glycoepitope‐expressing cases was higher in T4 than in T3 tumors (Fig. S1A). In addition to screening primary tumors, we also examined lymph nodes and distant metastases in 10 cases that presented sufficient tissue for analysis. We found a slightly higher number of tumors expressing sLeX (85% sLeX *vs* 76% sLeA) and that this antigen was present in 67% of lymph nodes (4/6) and 80% of distant metastases (8/10), irrespective of their origin (peritoneal or hepatic; Fig. S1B). Furthermore, sLeA showed expression in over 80% of both lymph node and distant metastases. Also, 80% of the metastases mimicked the expression of sLe antigens found in the primary tumor, reinforcing the close link between these sialoglycans and metastasis. However, no associations were found between the expression of these antigens, alone or in combination, with stage, prognosis, or presence of metastases among advanced stage patients. These findings may be directly linked to the nature of the patient sample set used for this study, which is mostly representative of advanced stage disease. We further devoted to analyzing healthy tissues with the aim of understanding the cancer specificity of these glycoepitopes (colon, skin, lung, stomach, pancreas, liver, appendix, thyroid, and testis; Fig. 1B). We found sLeA in colon goblet cells as well as the pancreas, goblet cells of the appendix, and the *stratum lucidum* of the skin. On the other hand, sLeX could not be detected in the colon but was present in the pulmonary alveoli and mucus secreting cells in the appendix. In white blood cells, sLeX was notably overexpressed, particularly in granulocytes (76% granulocytes; 15% lymphocytes; and 9% monocytes), showing a prevalence of 68%. In contrast, sLeA was expressed at a lower rate of 10%, predominantly in monocytes (65%) and lymphocytes (25%) when compared to its expression in granulocytes (9%; Fig. 1C, Fig. S2, Table S3). Furthermore, only a few cells co‐expressed both glycoepitopes (< 10%), mostly on monocytes (> 60%). The results were consistent across three different donors (Table S3). In summary, these findings reinforce the notion that sLe antigens exhibit low cancer specificity, particularly sLeX, which shows significant expression in leukocytes.

### 3.2 sLe glycogenes in CRC

**Fig. 2:**
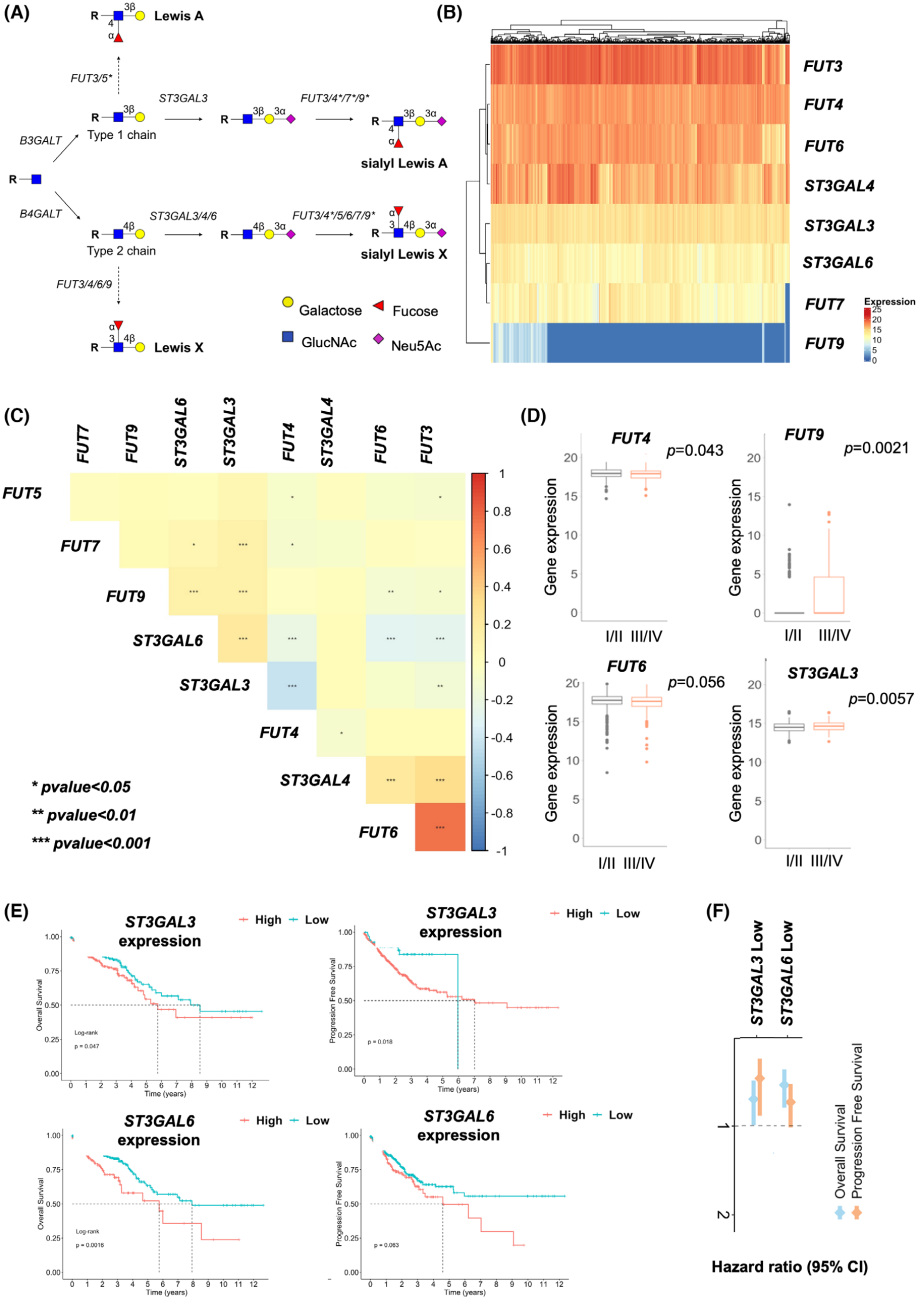
Colorectal cancer (CRC) exhibits two distinct sLe‐related glycogenes signatures linked to prognosis. (A) Schematic representation of the biosynthesis of sialyl Lewis A and X (sLeA and X) glycoepitopes. This schematic representation highlights the main biosynthesis routes for sLeA and sLeX terminal glycoepitopes, which are present in more elongated N‐ and O‐glycosidic chains in proteins as well as lipids. It particularly emphasizes the fucosyltransferases (*FUT3*, *4*, *5*, *6*, *7*, *9*) and sialyltransferases (*ST3GAL3*, *4*, *6*) involved in the modifications of Type 1 and Type 2 glycosidic chains that form the backbones of sLeA and sLeX. FUTs marked as “*” fucosylate *O*‐3 GlcNAc residues on Type 2 glycosidic chains, even though *O*‐4 fucosylation cannot be overulled. FUTs marked with “*” preferentially fucosylate *O*‐3 GlcNAc residues on Type 2 glycosidic chains, even though *O*‐4 fucosylation cannot be excluded. (B) Clustering analysis highlights the existence of two groups of *FUT*s and *ST3GALT*s driving sLe biosynthesis in CRC. CRC tumors segregate into two distinct groups based on the expression of glycogenes involved in sLe biosynthesis (*FUT3*, *FUT4*, *FUT6*, *ST3GAL4 vs FUT7*, *FUT9*, *ST3GAL3*, *ST3GAL6*). Notably, the expressions *FUT3*, *FUT4*, *FUT6*, *ST3GAL4* are markedly more elevated in CRC compared to *FUT7*, *FUT9*, *ST3GAL3*, *ST3GAL6*. (C) A correlation analysis supports the existence of two glycogenes groups in CRC. The correlogram shows significant positive correlations between *FUT3*, *FUT4*, *FUT6*, *ST3GAL4* and negative correlations with *FUT7*, *FUT9*, *ST3GAL3*, *ST3GAL6*, characterizing the second group of sLe‐related glycogenes. On the other hand, *FUT7* and *FUT9* correlated positively with *ST3GAL3* and *ST3GAL4* and negatively with the other group of glycogenes, reinforcing the existence of two distinct clusters of potentially co‐expressed glycogenes. Correlation analysis was performed using Spearman's rank correlation. (D) *FUT3* and *FUT4* were significantly decreased in more aggressive tumors (stages III/IV *vs* I/II), whereas *FUT9* and *ST3GAL3* transcripts were significantly increased. These observations link the group of tumors enriched for *FUT9* and *ST3GAL3* to cancer aggressiveness. Wilcoxon rank test was performed to assess differences between tumor stages. Error bars extend to 1.5 times the interquartile range (IQR) from the box edges. (E, F) Overexpression of *ST3GAL3* and *ST3GAL6* required for sLe antigens biosynthesis is associated with decreased survival in CRC. *ST3GAL3* and *ST3GAL6* overexpressing tumors presented significantly decreased overall and disease‐free survivals. Furthermore, tumors showing low transcripts for these glycogenes presented lower risk of death by cancer (*ST3GAL3*: HR = 0.7, *P* = 0.05; *ST3GAL6*: HR = 0.5, *P* = 0.002) and disease progression (*ST3GAL3*: HR = 0.5, *P* = 0.02; *ST3GAL6*: HR = 0.7, *P* = 0.06). *P* value obtained by log‐rank test and Cox Proportional Hazards Regression Analysis. Statistical significance was considered when *P* ≤ 0.05.

To gain more insights into the glycophenotype of CRC, we conducted a comprehensive analysis leveraging data from approximately 600 CRC cases, spanning all stages of the disease, along with 51 adjacent healthy mucosa samples retrieved from the TCGA database. Our investigation focused on key glycogenes, specifically fucosyltransferases (*FUT3*, *FUT4*, *FUT5*, *FUT6*, *FUT7*, and *FUT9*) and sialyltransferases (*ST3GAL3*, *ST3GAL4*, and *ST3GAL6*), known to play a direct role in the biosynthesis of sLe antigens (Fig. 2A). We found that *FUT4* and *FUT7* transcripts were elevated in CRC compared with healthy tissues, whereas the other glycosyltransferases were downregulated (Fig. S3). Notably, *FUT4* and *FUT7* are preferentially responsible for *O*‐3 fucosylation of type 2 chains leading to the formation of sLeX (Fig. 2A). On the other hand, *FUT3* linked to *O*‐4 fucosylation was downregulated in CRC, suggesting a predominance of type 2 over type 1 fucosylated structures, thus in accordance with immunohistochemistry analyses (Fig. 1A). *FUT5* was residually expressed in few healthy and CRC cases and therefore disregarded in future evaluations. Focusing on glycosyltransferases expression in tumor tissues, we observed two distinct expression clusters, segregating *FUT3*, *FUT4*, *FUT6*, and *ST3GAL4* transcripts, from *FUT7*, *FUT9*, *ST3GAL3*, and *ST3GAL6* (Fig. 2B). Correlation analysis further reinforced these observations (Fig. 2B,C). Accordingly, one group showed strong positive correlations between *FUT3*, *FUT6*, and *ST3GAL4*, along with negative correlations with *FUT9*, *ST3GAL3* and *ST3GAL6* (Fig. 2C). The other group presented the opposite phenotype, with *FUT9* and *ST3GAL3* being significantly correlated positively with *ST3GAL6* and negatively with *FUT4*. In addition, *ST3GAL3* also correlated positively with *FUT7* and negatively with *FUT3*. Collectively, these findings reinforce the existence of two distinct groups of glycosyltransferases (*FUT3*, *FUT4*, *FUT6*, *ST3GAL4 vs FUT7*, *FUT9*, *ST3GAL3*, *ST3GAL6*) potentially responsibly by sLe antigens biosynthesis in CRC, suggesting patterns of co‐expression or regulatory relationships among them. Finally, we analyzed their expression according to the severity of disease by comparing early (stage I/II) with late stage (III/IV) tumors. Despite differences between cancer and healthy tissues, most glycosyltransferases' expression remained unchanged with disease stage (data not shown). The exceptions were *FUT9* (*P* = 0.0021) and *ST3GAL3* (*P* = 0.0057), which were statistically overexpressed in advanced (III/IV) compared with early‐stage tumors (I/II; Fig. 2D), whereas *FUT4* (*P* = 0.043) and *FUT6* (without reaching statistical significance, *P* = 0.056) were downregulated (Fig. 2D). In agreement with these observations, we found *ST3GAL3* and *ST3GAL6* overexpression to be associated with worse prognosis, translated by decreased overall and disease‐free survivals (Fig. 2E). Furthermore, tumors exhibiting low *ST3GAL3* expression had a hazard ratio (HR) of 0.7 for overall survival (*P =* 0.05) and 0.5 for disease‐free survival (*P* = 0.02; Fig. 2F). Similarly, for low *ST3GAL6* expression, the HR was 0.5 for overall survival (*P* = 0.002) and 0.7 for disease‐free survival (*P* = 0.06; Fig. 2F). Conversely, tumors overexpressing *FUT3*, *FUT4*, and *FUT6* showed better prognosis (Fig. S4). Tumors showing a downregulation of these glycosyltransferases exhibited significantly higher probabilities of cancer related death and disease progression (*FUT3*low: HR‐1.8, *P* = 0.04 for overall survival/HR‐1.7, *P* = 0.04 for progression free survival; *FUT4*low: HR‐2.4, *P* = 0.02/HR‐1.8, *P* = 0.002; *FUT6*low: HR = 1.6, *P* = 0.006/HR‐1.6, *P* = 0.01; *FUT9*low: HR = 0.7, *P* = 0.09/HR‐0.7, *P* = 0.19; Fig. S4). Given the clear link between *ST3GAL3* overexpression and poor prognosis, we further examined its expression in a subset of advanced human tumors (stage III/IV) with varying levels of sLe antigens. This analysis confirmed that tumors overexpressing sLe antigens had significantly higher levels of *ST3GAL3* mRNA compared to those not expressing these antigens (Fig. S5), reinforcing ST3GAL3's key role in the biosynthesis of sLe antigens in advanced CRC. In summary, we found two distinct glycosyltransferases expression patterns linked to different prognoses. Namely, we found indications that elevated *ST3GAL3* and *ST3GAL6* transcripts may be used as surrogates of poor prognosis, underscoring a connection between hypersialylation driven by these glycosyltransferases and CRC aggressiveness. Their strong correlation with *FUT7* and *FUT9* supports the preferential expression of sLeX antigens in more aggressive tumors, aligning with our observations in patient samples. On the other hand, we found the high *FUT3*, *FUT4*, and *FUT6* transcripts were surrogates favorable prognosis. Our findings further highlight the importance of gaining a deeper understanding of the molecular pathways underlying sLe biosynthesis and accurate mapping of these terminal sialoglycans through glycomics, particularly in the context of precision oncology.

### 3.3 E‐selectin affinity glycoproteome

**Fig. 3:**
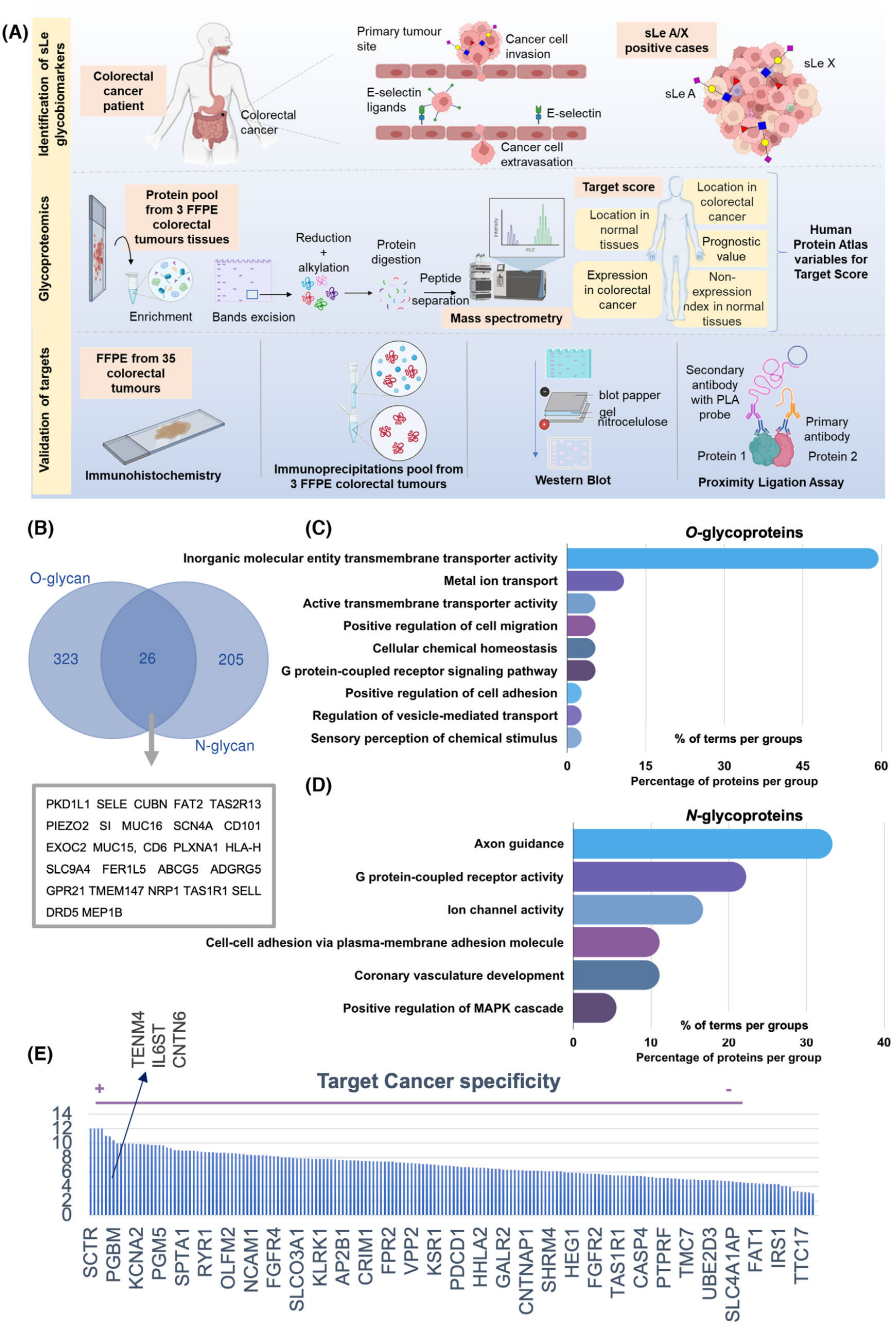
E‐selectin affinity glycoproteomics identifies relevant functional molecular signatures and targetable glycoproteins potentially carrying sialylated Lewis antigens. (A) Schematic representation of the analytical workflow explored for identification of E‐selectin ligands in colorectal cancer (CRC). The top panel illustrates how sialylated Lewis antigens may facilitate the intravasation and extravasation of cancer cells through interactions with E‐selectin on endothelial cells. The middle panel outlines the workflow for the isolation and identification of glycoproteins, along with the curation of glycoproteomics data to identify potentially targetable molecular signatures. In brief, protein extracts from Formalin‐fixed paraffin‐embedded (FFPE) metastatic CRC tissues were pooled, enriched for sialylated Lewis antigens expressing glycoproteins through affinity for E‐selectin and separated by SDS/PAGE. The bands were excised, and the proteins were reduced, alkylated, and digested with trypsin before being analyzed by nanoLC‐ESI‐HCD‐MS/MS. Identified glycoproteins were ranked using an in‐house algorithm (target score), which utilizes information from the Human Protein Atlas to sort proteins based on their targetability, by summing cancer specificity and association with poor prognosis, while penalizing expression in healthy tissues. Relevant glycoproteins were then orthogonally validated in cancer tissues for sLe antigen expression using different immunoassays such as immunohistochemistry, proximity ligation assay (PLA), and western blot. Different elements that constitute this panel were obtained from Biorender.com. (B) More O‐glycoproteins potentially carrying sialylated Lewis antigens were identified compared to N‐glycoproteins. Notably, only 26 glycoproteins were found carrying both modifications. (C, D) The most notable distinction in terms of biological functions between the O‐ and N‐glycoproteomes was the association of N‐glycoproteins with neuronal traits. GO term analysis showed that O‐glycoproteins were mainly involved in plasma membrane molecular transport and cellular homeostasis regulation, as well as in cell adhesion, migration, and G protein‐coupled receptor signaling pathways. Furthermore, proteins linked to sensory functions suggest an association with neuroendocrine functions, mirroring observations for N‐glycoproteins. N‐glycoproteins are primarily associated with axon guidance, and to a lesser extent, with G protein‐coupled receptor and ion channel activities, as well as cell adhesion, similarly to O‐glycans. Notably, glycoproteins involved in vasculature development and positive regulation of the MAPK cascade were also observed. (E) Secretin receptor (SCTR) emerged as one of the top ranked targetable glycoprotein in CRC. Most abundant glycoproteins were ranked using our in‐house target score algorithm, which builds on the Human Protein Atlas database to ascertain cancer specificity and associations with poor prognosis. Notably, this approach identified SCTR, along with TENM4, IL6ST, and CNTN6, as top‐ranked glycoproteins.

To overcome the limitations arising from the noncancer specificity of sLe antigens, we undertook a thorough characterization of the glycoproteome to pave the way for precision oncology (according to the scheme in Fig. 3A). Our approach involved using proteins extracted from FFPE tissues as the starting material. The goal was to highlight the underexplored yet promising potential of this material for conducting comprehensive glycome and glycoproteome studies. A pool of three metastatic tumors overexpressing sLeA/X in more than 50% of the tumor area was selected for a thorough investigation of the sLe‐glycoproteome. This involved utilizing E‐selectin affinity chromatography followed by bottom‐up identification through mass spectrometry (Fig. 3A). Glycomics analysis, involving the *in situ* N‐ and O‐deglycosylation of sLe hotspots in tumor tissues, was conducted in parallel to facilitate the annotation and validation of glycoproteins. We began by observing relevant similarities between the N‐glycomes across the three studied samples, as reflected by the coefficient of variation for the most abundant glycan structures (Fig. S6A, Table S4). Complex N‐glycans emerged as the most expressed class of glycans in these samples, comprising approximately 60% of all identified species, followed by 22% oligomannoses (Fig. S6A, Table S4). In contrast, the O‐glycome was mainly composed of high amounts of mono‐ and di‐sialylated T antigens (compositions: H1N1S1; H1N1S2; Fig. S6B, Table S5) but showed a higher degree of heterogeneity across samples in terms of relative glycan abundances (Table S5). Another noticeable difference was the very low expression of sialylated core 3 (N2S1; Table S5) in one of the samples. These observations support the need for deeper investigation into glycome heterogeneity. Nevertheless, glycan structures potentially compatible with sialylated Lewis antigens were more prevalent among N‐glycans (approximately 18% of total N‐glycans; Table S4) compared with O‐glycans (> 10%; Table S5). These structures were mainly observed on complex N‐glycans (H4N4F1S1; H5N4F1S1; H5N4F1S2; H6N5F1S1) and in extended core 1 and 2 O‐glycans (H1N1S1F1; H1N2S1F1; H2N2S1F1; H2N2S2F1; Fig. S6A,B), also in accordance with previous reports on the CRC glycome [5]. The presence of sLe antigens on N‐ and O‐glycans was further confirmed by MS/MS analysis (Fig. S6C,D). These data were later used for unequivocal identification of sLe glycoproteoforms by mass spectrometry.

We have identified 554 glycoproteins carrying glycopeptides with masses suggesting the presence of sLe antigens (Fig. 3B, Tables S6 and S7). This list only included proteins with glycopeptides showing spectra with oxonium ions for hexosamine, sialic acids and the disaccharide composed of an hexosamine‐hexose. Notably, the number of O‐glycoproteins (*n* = 349; Table S6) potentially carrying sLe glycoepitopes was higher than that of N‐glycoproteins (*n* = 231; Table S7). However, it was only possible to ascertain the simultaneous occurrence of both types of glycosylation in 26 proteins. These included mucins (MUC‐15; MUC‐16; Fig. 3B) and proteins associated with cell adhesion, such as FAT2 (commonly found in the nervous system) [22] and CD6, which may serve as a costimulatory molecule for T‐cell activation and proliferation [23]. Additionally, adhesion‐related molecules such as E‐selectin and L‐selectin, typically associated with lymphocytes, hinted at the possibility that these receptors may also carry sLe antigens. Nevertheless, bleeding from the enrichment strategy cannot be overruled at this stage. The comprehensive integration of these data using GO term analysis revealed markedly different biological functions (Fig. 3C,D). Identified O‐glycoproteins predominantly play a crucial role in facilitating the transport of inorganic molecules and ions across the cell membrane, accounting for over 70% of their involvement (Fig. 3C). They also contribute, albeit to a lesser extent, to cellular functions such as adhesion, migration, chemical homeostasis, and participation in the G‐protein coupled receptors (GPCRs) signaling pathway (Fig. 3C). In contrast, the N‐glycoproteins carrying sLe epitopes reveal distinct molecular networks (Fig. 3C). Beyond mediating GPCRs and ion channel activities, as well as cellular adhesion, these proteins are associated with vasculature development and positive regulation of the Mitogen‐Activated Protein Kinase (MAPK) cascade, a pathway linked to promoting proliferation, resistance to apoptosis and poor prognosis in CRC [24, 25]. Notably, the most significant and markedly different biological function is related to axon guidance (> 30% of the glycoproteins; Fig. 3D). This involves the processes through which developing nerve cells navigate and extend their axons to establish connections within the nervous system. Interestingly, a more detailed revisitation of the identified N‐ and O‐glycoproteins, supported by the Human Protein Atlas database, identified several protein species typical of neural cells, namely CNTN6, NRCAM, NFASC, TENM4, and NCAM1. The presence of glycans carrying sLe antigens on these glycoproteins was further confirmed by manual annotation of MS/MS spectra (Fig. S7). These observations strongly suggest that these sialoglycoproteins may originate from the neuroendocrine system, constituting novel findings that warrant more in‐depth confirmation in future studies. Interestingly, in explorative settings, we observed that all three tumors pooled for this study presented significant vascularization (high CD34; Fig. S8), and one also displayed high levels of the neuroendocrine marker synaptophysin (Fig. S8). Such observations align with the connection between the identified glycoproteins, angiogenesis, and neural features. Another noteworthy discovery was the identification of the immune checkpoint PD‐1, now being described as an E‐selectin binder for the first time. However, the implications of these findings for protein functionality as well as its clinical significance remain unaddressed and merit future investigations. We then compared our findings with the limited information on E‐selectin ligands from CRC cell lines [5, 26] (Fig. S9). While the number of glycoproteins identified by our study significantly broadens the understanding of the E‐selectin affinity CRC glycoproteome compared with the current literature, we were able to identify some common grounds with a recent investigation on a sLeX‐expressing cell line [26] (Fig. S9). We particularly highlight nervous system‐associated glycoprotein L1CAM, which suggests that cells of epithelial origin may gain capacity to express neuronal markers carrying sLe antigens.

### 3.4 Target selection

Finally, we have conducted a thorough exploitation of the identified glycoproteins to uncover potential targetable signatures, considering cancer specificity and prognostic links. This comprehensive analysis utilized an in‐house algorithm, previously successful in identifying cancer‐specific glycoproteoforms in esophageal [8], gastric [9], and bladder cancer [10]. The algorithm systematically utilizes the protein databank to prioritize associations with cancer, poor prognosis, preferential localization at the cell membrane for enhanced targetability, and low expression in relevant healthy organs to minimize potential off‐target effects. Conversely, the algorithm‐imposed penalties for the presence of glycoproteins in healthy tissues. Accordingly, the most abundant glycoproteins were then analyzed by this approach. Notably, among the top ranked glycoproteins carrying sLe antigens was TENM4 and the SCTR (Fig. 3E, Table S8). Interestingly, both glycoproteins have been observed in nerve cells [27, 28].

### 3.5 SCTR and SCTR sialoproteoforms in CRC

**Fig. 4:**
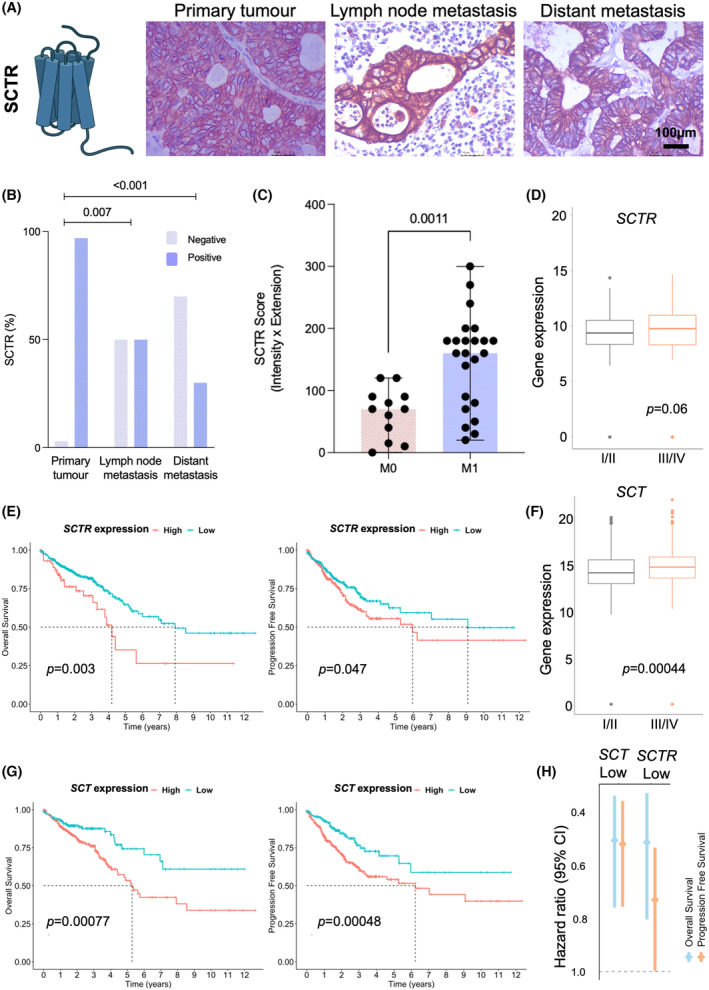
The secretin receptor (SCTR) is overexpressed in metastatic tumors as well as in the metastases and associates with poor prognosis in colorectal cancer (CRC). (A, B) The SCTR is highly expressed in both aggressive primary tumors as well as lymph node and distant metastases. The SCTR exhibits diffuse expression in most colorectal tumors and may also be present in some lymph nodes (in 50% of cases) and distant metastases (in 25% of cases), predominantly in the liver. Notably, no signs of SCTR expression were observed in peritoneal metastases. A Chi‐square test was used to compare the expression levels of SCTR between the groups. (C) The SCTR is significantly more expressed in primary tumors showing metastasis. Wilcoxon rank test was used to compare the expression of SCTR in non‐metastatic (M0) vs metastatic tumors (M1). Mean ± SD (standard deviation). (D) The *SCTR* gene is overexpressed in advanced stage CRC. The Cancer Genome Atlas (TCGA) analysis showed that the number of *SCTR* transcripts was higher for advanced tumors (stages III/IV vs I/II). Differences on the expression of *SCTR* transcripts among clinical stages was assessed using Wilcoxon rank tests. Variation of the box and whisker plot restricted to 1.5 times the interquartile range (error bars). (E) *SCTR* overexpression associates with decreased survival. Tumors exhibiting *SCTR* overexpression showed significantly lower overall and progression‐free survivals compared to tumors with low *SCTR* expression. Kaplan–Meier analysis and log‐rank test were used to compare overall survival (OS) and progression‐free survival (PFS) curves. (F) Secretin (*SCT*) is also overexpressed in advanced stage tumors. Advanced stage tumors present significantly higher number of *SCT* transcripts, which encode for a key activating ligand of SCTR. Wilcoxon rank tests was performed to assess the difference between the two groups. The error bars in the boxplot extended to 1.5 times the interquartile range (IQR) from the box edges. (G) *SCT* overexpression associates with worse survival. Tumors exhibiting high *SCT* transcript levels present significantly lower overall and disease‐free survival rates when compared to *SCT*‐low tumors. OS and PFS curves were compared using Kaplan–Meier analysis and log‐rank tests. (H) Low *SCTR* or *SCT* transcript levels are associated with a decreased risk of death. Statistical significance was considered when *P* ≤ 0.05.

**Fig. 5:**
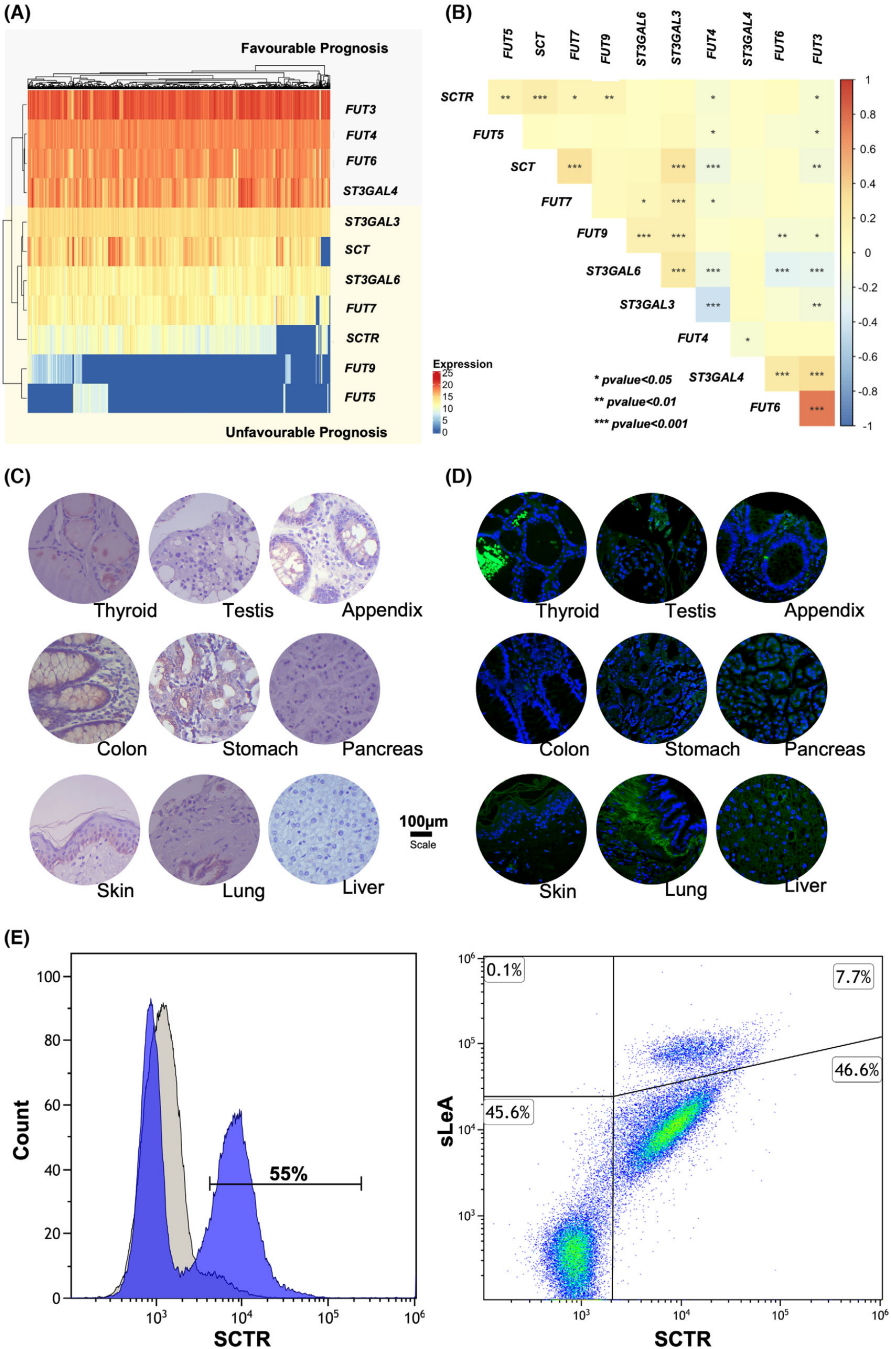
Secretin receptor‐sialyl Lewis (SCTR‐sLe) glycoproteoforms associate with worst prognosis and present a very restricted pattern in human healthy tissues and cells. (A) The SCTR and its activating ligand secretin (SCT) cluster with sLe‐related glycogenes linked to worst prognosis in colorectal cancer (CRC). *SCTR* and *SCT* expressions clusters with the expression of focusyltranferases *5*, *7*, *9*, *ST3GAL3* and *ST3GAL4* linked to worst prognosis in CRC. (B) The SCTR is significantly correlated with SCT and the glycogenes linked to worst prognosis. The SCTR receptor positively correlates with SCT and glycogenes linked to a poor prognosis, while both are negatively correlated with the glycogenes associated with a favorable prognosis. Correlation analysis was performed using Spearman's correlation. (C) SCTR presents relevant expression in several human healthy organs (respiratory system; gastrointestinal tract; pancreas; kidney and urinary bladder). The analyses of healthy organs revealed moderate SCTR expression in the healthy glandular epithelium of the gastrointestinal tract, specifically in the stomach, colon, and appendix, as well as in the bronchi and bronchioles. (D) SCTR‐SLeA glycoproteoforms were not detected in healthy tissues. PLAs conducted on healthy tissues, including SCTR‐positive/SLeA tissues (colon, pancreas, skin) did not reveal signs of proximity, consistent with the observation that the glycoepitope and the SCTR are not expressed in the same region. (E) SCTR‐sLeA glycoproteoforms are residually expressed by circulating leukocytes. SCTR is highly expressed in leukocytes, with approximately 55% of them showing expression, primarily in granulocytes (75%) and some lymphocytic populations and monocytes. Additionally, a residual population of approximately 8% of monocytes expresses both SCTR and sLeA.

**Fig. 6:**
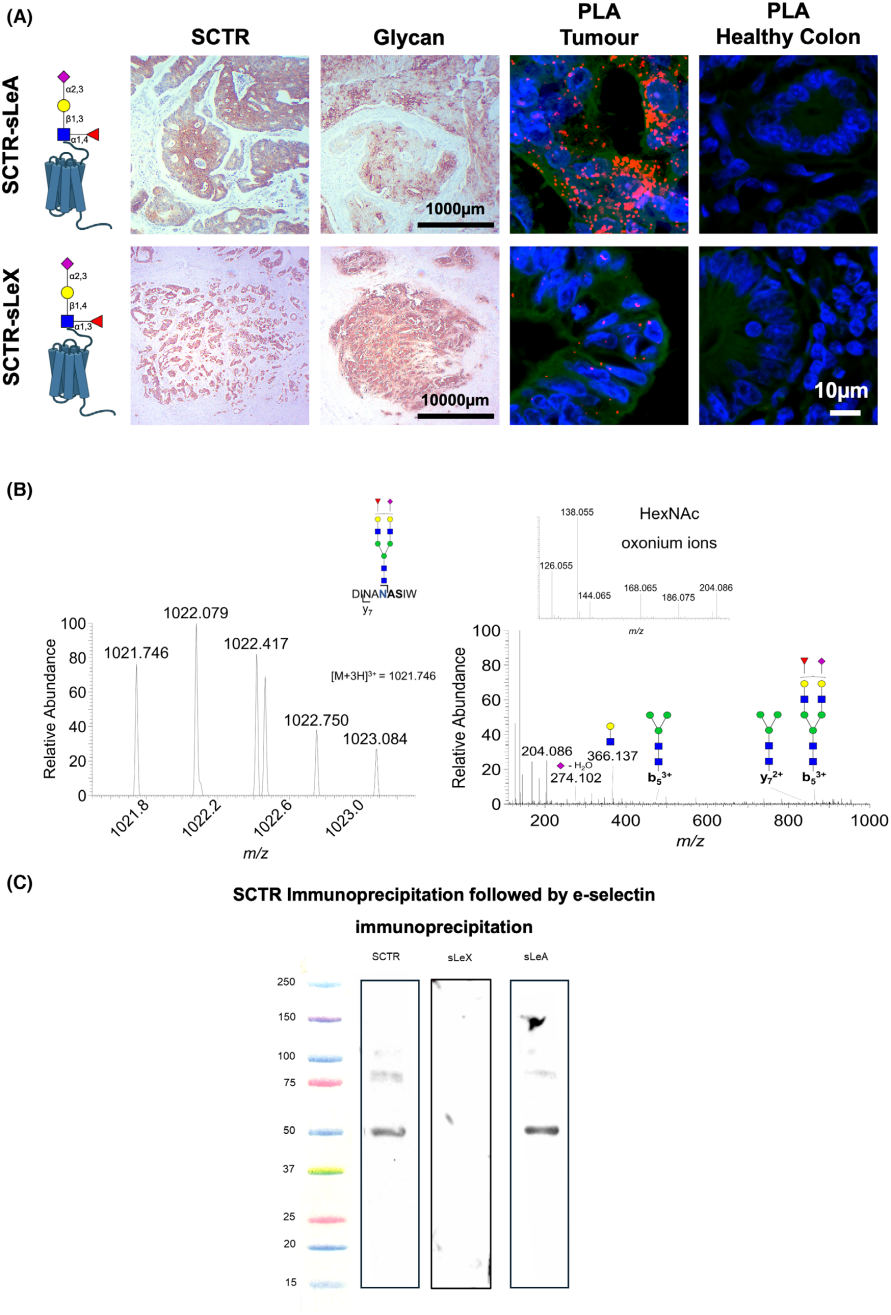
The SCTR is a major carrier for sialylated Lewis antigens in CRC but not on healthy tissues. (A) The Secretin Receptor (SCTR) predominantly carries sialyl Lewis A (sLeA) in CRC. Immunohistochemistry analysis revealed a co‐expression of both sLeA and sLeX (sialyl Lewis X) glycoepitopes along with SCTR in the same tumor area, which was not observed in healthy tissues denoting some degree of cancer specificity. Proximity ligation assay (PLA) analysis further confirmed SCTR as a potential carrier for these glycans, a finding absent in healthy colon samples. Moreover, PLA analysis strongly suggests that SCTR is predominantly modified with glycans carrying sLeA. (B) Full mass spectrum (MS) and fragmentation mass spectra for an SCTR N‐glycopeptide potentially carrying sialylated Lewis antigens. The MS and MS/MS spectra support the existence of a complex type N‐glycan potentially carrying sialylated Lewis antigens. Collectively, multiple orthogonal validations support the modification of SCTR with glycans carrying sialylated Lewis antigens, most likely sLeA. (C) Western blot analysis of SCTR‐immunoprecipitates provides evidence for the existence of SCTR‐sLeA glycoproteoforms acting as ligands for E‐selectin. Briefly, SCTR was first immunoprecipitated from protein extracts isolated from a pool of metastatic CRC FFPE tissues used for glycoproteome characterization. The immunoprecipitation (IP) product was then separated in a second dimension by E‐selectin affinity chromatography to enrich for sLe‐expressing glycoproteoforms. Western blot analysis showed a main band above 50 kDa, a less intense band below 100 kDa, and a faint band between 100 and 120 kDa, all reactive for SCTR. A similar pattern was observed when using an anti‐sLeA antibody, but not with the anti‐sLeX antibody, supporting sLeA as the main glycoepitope on these glycoproteoforms.

In this study, we focused on assessing the cancer specificity and the clinical relevance of the SCTR in CRC, aiming to gain more information about this protein, which has been poorly studied in this context. This membrane glycoprotein was expressed in all primary advanced cases (Fig. 4B), diffusely expressed across the tumor tissue (> 70% of the tumor area). It was also significantly overexpressed in lesions showing distant metastasis (Fig. 4C). Notably, the SCTR was It was also detected in 50% of lymph node metastases (3/6) and 30% of distant metastasis (3/10; Fig. 4B, Fig. S10). Strikingly, all distant positive metastases were observed in the liver, suggesting some degree of tropism. Despite being expressed in relevant number of metastases, the SCTR was predominantly present in primary tumors (Fig. 4B). Complementary transcript analysis from TCGA confirmed the observations made in our patient series, linking the *SCTR* receptor with CRC aggressiveness. The results suggest a possible trend of increased *SCRT* in more advanced stage III/IV, although not statistically significant (*P* = 0.06; Fig. 4D). Also, *SCTR* overexpression was associated with decreased overall (*SCTR* ^low^ tumors: HR = 0.5; *P* = 0.004) and progression‐free (*SCTR* ^low^ tumors: HR = 0.7; *P* = 0.05; Fig. 4E–H) survivals in CRC. Similar behavior was also observed within more advanced stage cases (Fig. S11), thus consistent with observations made in our patient series involving this patient subset. Interestingly, TCGA analysis also unveiled a significant positive correlation between *SCTR* and *SCT* for a relevant subset of colorectal tumors (Fig. 5B). Notably, SCT binding to the SCTR receptor has been found required for initiating physiological responses [29]. Furthermore, *SCT* was also significantly overexpressed in stage III/IV CRC and associated with worse prognosis (overall survival for *SCT* ^low^ tumors HR = 0.5, *P* = 0.001; disease‐free survival HR = 0.5, *P* = 0.001; Fig. 4F–H). Notably, clustering analysis revealed an association between SCRT and SCT with glycosyltransferases linked to the unfavorable prognosis glycosyltransferase phenotype. (Fig. 5A). These findings were further reinforced by correlation analysis, where *SCTR* and *SCT* expressions correlated positively with *FUT4*, *5*, and *9* linked to the worst prognosis (Fig. 5A,B). Conversely, *SCRT* correlates negatively with *FUT3* and *FUT4*, associated with favorable prognosis (Fig. 5A,B). *SCT* expression correlated positively with *FUT7* and negatively with *FUT3* and *FUT4*. Additionally, it correlated positively with poor prognosis marker *ST3GAL3* (Fig. 5B). Finally, we analyzed a subset of advanced tumors (stage III/IV) from the TCGA database, which more closely matched the patient samples from our hospital used for the initial SCTR screening. We found that tumors overexpressing SCTR had decreased overall survival (*P* = 0.036), although there was no clear association with progression‐free survival in these cases (Fig. S11). In addition, *SCT* overexpression was also associated with worst survival in advanced CRC (OS: *P* = 0.0013; PFS: *P* = 0.041; Fig. S11). Collectively, these findings reinforce a link between SCTR and SCT cancer aggressiveness and sLe antigens driven by a particular subset of glycosyltransferases, which merits future validation. We then assessed the glycosylation of SCTR, aiming to determine cancer specificity. Approximately 70% of the cases positive for SCTR, either tumors or metastases, coexpressed sLe antigens in the same tumor area (Fig. 6A). PLAs targeting sLeA and sLeX provided additional support for the hypothesis that SCTR could function as a carrier for these glycoepitopes (Fig. 6A). Furthermore, PLA analysis of the same tumor tissue sections, postdigestion with PNGase F to eliminate N‐glycans, led to the disappearance of the SCTR‐sLe signals (data not shown). The hypothesis that sLe antigens could be attached to N‐glycans was further supported by tandem mass spectrometry, revealing glycopeptides potentially carrying complex N‐glycans terminated by sLe antigens (Fig. 6B). PLA analysis further showed a higher density of SCTR‐sLeA in comparison with the SCTR‐sLeX glycoproteoform in CRC (Fig. 6A). For orthogonal validation, we immunoprecipitated SCTR from protein pools of metastatic CRC used for glycoproteomics. We then isolated glycoproteoforms with affinity for E‐selectin, using the same method applied for sLe‐glycoprotein enrichment. The SCTR western blot showed a main band above 50 kDa, a less intense band below 100 kDa, and a faint band between 100 and 150 kDa, likely corresponding to highly glycosylated proteoforms (Fig. 6C). The blot for sLeA showed a similar pattern, whereas no reactivity was observed for sLeX, thus consistent with *in situ* PLA. Taken together with immunoassays in patient samples (Fig. 6A–C) and mass spectrometry (Fig. 6B), these findings reinforce that SCTR is a carrier for sialylated Lewis antigens. Additionally, we have demonstrated that, despite the high expression of sLeX in CRC cancer, the receptor is a preferential carrier for sLeA and can be targeted by E‐selectin, potentially explaining its presence in metastatic contexts.

### 3.6 SCTR and SCTR sialoproteoforms in healthy tissues

Finally, we conducted a more in‐depth investigation on the cancer specificity of the SCTR exploiting documented analysis in the human protein atlas. The receptor has been found highly expressed in the gastrointestinal tract and, to a lesser degree, in the respiratory system as well as in the pancreas, kidney, and urinary bladder. Low expressions could also be found in proximal digestive tract, in male, connective and soft tissues (Fig. S12). Additionally, and for validation purposes, we also analyzed the cohort of healthy tissues previously screened for sLe antigens. We confirmed the expression of SCTR across the digestive tract (stomach, colon, and appendix; Fig. 5B) in cells of the glandular epithelium as well as in respiratory tract in cells that compose the bronchus and bronchiole epithelia in all studied samples. The thyroid, testicle, liver, and pancreas were negative (Fig. 5B), which contrasted with the low expression described by the human protein atlas. However, despite the expression of sLe antigens in some of these tissues (lung, pancreas, colon, and appendix, skin; Fig. 1B), we found no indications of overlapping with SCTR‐sLe expressing areas, a finding later confirmed by negative PLA (Fig. 5C). Furthermore, we identified SCTR in around 50% of leukocytes, predominantly present in granulocytes (approximately 75%), and to a lesser extent, in 15% of lymphocytes and 10% of monocytes (Fig. 5D, Fig. S13, Table S9). However, less than 10% of leukocytes also exhibited positivity for sLeA, with little variations across the three different donors. Interestingly, the distributions among granulocytes and lymphocytes across different donors was considerably variable (Fig. 5D, Fig. S13, Table S9), denoting some degree of intersubject variability that warrants further investigations. In summary, our findings indicate a constrained expression pattern for SCTR‐sLeA glycoproteoforms in healthy tissues, in clear contrast with their elevated presence in colorectal primary tumors and metastases.

## 4 Discussion

This work establishes a roadmap for identifying glycoproteoforms carrying sialylated antigens in CRC, with potential applicability to other tumors. The study was motivated by the crucial role played by these glycans driving metastasis [30, 31]. They facilitate cancer cell engagement with endothelial cells, aiding intravasation, enabling travel through the bloodstream, and ultimately directing them to distant locations via interactions with E‐selectin [3, 32]. In fact, blocking cancer cell binding to E‐selectin has been demonstrated to prevent CRC metastasis [33, 34]. However, this binding is influenced not only by the structural characteristics of the terminal sLe epitope but also by the protein scaffold carrying these post‐translational modifications [35, 36]. Still, the identity and functions of glycoproteoforms carrying these glycoepitopes in CRC and other tumors remain mostly elusive, hindering a comprehensive understanding of their biological roles and precision oncology. In fact, most studies concerning CRC have been exclusively conducted on cancer cell lines, leading so far to the identification of a limited number of glycoproteins such as CD44 [37], carcinoembryonic antigen [38], podocalyxin‐like protein [39], lysosomal membrane glycoprotein‐1 and 2 [32], and several types of integrins [26]. The low cancer specificity of sLe antigens also poses a limitation for targeted therapies.

To address these challenges, we applied E‐selectin affinity chromatography and tandem mass spectrometry, a methodology successfully employed in breast [16], gastric [9], and CRC cell models [26] to analyze glycoproteomics in tumor tissues. Notably, FFPE tissues, predominant in cancer biobanks, proved invaluable for glycome and glycoproteome characterization, expanding beyond recent demonstrations on CRC glycome characterization in these tissues [5, 40]. Our investigation revealed a broader spectrum of CRC glycoproteins with affinity for E‐selectin compared to previous reports in cell models [26, 37]. We observed significant differences between the O‐ and N‐glycoproteomes in terms of identified glycoproteoforms and key biological functions. Crucial glycoproteins were identified for cell adhesion, maintenance of cellular homeostasis, response to microenvironmental stimuli, and activation of relevant oncogenic pathways, particularly MAPK cascade [41, 42]. We also unveiled a significant number of glycoproteins associated with neovascularization and introduced novel observations regarding the substantial presence of glycoproteins characteristic of the colorectal enteric nervous system, recognized for autonomously regulating physiological functions [43] but poorly characterized in CRC. Among these glycoproteins is the neuron‐associated glycoprotein LICAM1, also described in a recent study on the *FUT6*‐transfected sLeX‐overexpressing SW620 cell line as a major E‐selectin binding molecule [26]. LICAM1, characteristic of nervous cells, has been implicated in neuronal migration, neurite fasciculation, and synaptic plasticity, observed in several types of tumors, including CRC [44, 45, 46]. Its identification and overexpression, promoted by *FUT6*/sLeX in an adenocarcinoma cell line with epithelial morphology [26], pose a provocative hypothesis. It suggests that CRC cancer cells may acquire neurological traits potentiated by sLe, emphasizing the need for further confirmation. Our study demonstrates that glycosylation with sLe antigens is a molecular feature shared by glycoproteins associated with vascular and neuro functions, reinforcing potential yet undetermined links between these two systems. Additionally, we identified PD‐1, a well‐known immune checkpoint receptor targeted in clinics to enhance anti‐cancer immune responses [47, 48]. While PD‐1 N‐glycosylation is critical for receptor functionality and recognition by therapeutic antibodies [49, 50], the presence of sLe antigens is being reported for the first time, and its biological role remains unknown. Nevertheless, these findings strongly indicate the presence of T cells in the tumor bulk carrying sLe antigens, known to play a key role in T‐cell recruitment to inflammation sites by enabling adhesion to E‐selectin [51, 52]. Overall, we hypothesize that the diversity found in the sLe glycoproteome could potentially arise from shared microenvironmental pressures affecting different types of cells (immune, vascular, neuronal, and tumor) within the tumor ecosystem. Specifically, it is well‐established that sLe antigens expression may be triggered by pro‐inflammatory signals in various conditions, including cancer [53, 54]. In chronic gastric inflammation induced by *H. pylori*, the expression of sLe antigens has been linked to the modulation of glycosyltransferases by pro‐inflammatory cytokines, namely TNF‐α [55]. Additionally, in a primary invasive ductal carcinoma cell line, the inhibition of fucosylation suppressed sLeX antigen and E‐selectin ligands expression, resulting in lower migration capacity and reduced IL‐8 and TGF‐β expressions [41]. Furthermore, *FUT6* silencing has been shown to reduce TGF‐β‐mediated epithelial‐mesenchymal transition and inhibit migration in CRC [56]. These findings highlight the pivotal role of the microenvironment on the expression of sLe antigens. Future studies should explore the functional impact of these seminal observations and their consequences for organ functionality and disease progression. Emerging single cell proteomics approaches will be unvaluable tools toward this objective [57, 58, 59].

Finally, we have conducted a thorough analysis of identified glycoproteins to uncover potential targetable signatures using an in‐house algorithm successful in various cancers [8, 9, 10]. The G protein‐coupled SCTR emerged as a top‐ranked glycoprotein and was selected for in‐depth evaluations due to its relatively unexplored nature in CRC. Notably, the SCTR has been previously identified in both glandular cells and subsets of putative secretomotor/vasodilator neurons in the healthy colon [60]. This reinforces the hypothesis that many sLe‐expressing glycoproteins may originate from the peripheral nervous system. While it remains poorly studied in CRC, the SCTR has been found overexpressed in gastrinomas [61], bronchopulmonary carcinoid tumors [62] cholangiocarcinoma [61], esophageal, and pancreatic ductal adenocarcinomas [61] and considered a candidate target for molecular tumor imaging as well as for peptide receptor radioligand therapy [63]. Interestingly, esophageal [64] and pancreatic tumors [65] have been also previously described to overexpress sLeA, suggesting possible associations. Here, we describe that both SCTR and its activating ligand SCT are overexpressed at advanced stages CRC and associated with distant metastases, being predictors of worse prognosis. The correlation between elevated SCTR and SCT levels in CRC may imply yet unknown alterations in fluid balance, colonic motility, and interactions with the enteric nervous system, with implications for disease progression and dissemination. These constitute novel observations raising fundamental questions about SCTR's role in CRC biology. Furthermore, we have verified SCTR as a primary carrier of sLe antigens, proposing that these glycoepitopes might confer cancer specificity to the glycoprotein and enable adhesion to E‐selectin. However, further in‐depth analysis is essential to validate this hypothesis, evaluate the impact of glycosylation on SCTR functionality, and explore targeted therapeutic opportunities. Interestingly, we also linked the SCTR and SCT to a group of sLe‐related glycogenes defining worst prognosis, reinforcing the association with CRC aggressiveness. Furthermore, we have uncovered a novel clinical context and a potential coregulatory mechanism among sialyl Lewis‐related glycogenes in CRC, providing novel insights to better understand their biological functions and role in disease, which remain controversial.

## 5 Conclusions

This study presents a strategy for unraveling the cancer glycoproteome's complexity, identifying clinically relevant signatures, and shedding new lights on the sialoglycoproteome's links to the colonic peripheral nervous system. The recently recognized impact of nerves and neoinnervation on various cancer types [66, 67, 68] underscores the imperative need for deeper exploitation. Specifically, while interactions between cancer, vascular, and nervous cells have been shown to contribute to cancer progression [69, 70], the precise mechanisms driving CRC and the role played by protein glycosylation in this context remain unexplored. We anticipate that this knowledge may provide a novel research avenue at the intersection of neurology and oncology, paving the way for precision oncology and novel strategies to counteract metastases development. Furthermore, our investigation revealed an overexpression of SCTR carrying sLeA and targeted by E‐selectin in metastatic colorectal tumors, as well as a significant number of associated lymph node and distant metastases. Notably, this overexpression contrasts with the restricted expression pattern observed in healthy human tissues and immune cells. While the possibility of cancer specificity remains, further studies leveraging a comprehensive characterization of the human glycoproteome are essential. Nonetheless, these findings provide a foundation for targeted approaches to address aggressive CRC tumors and metastases more effectively.

## Conflict of interest

The authors declare no conflict of interest.

## Author contributions

Conceptualization was taken care by SC, DF, and JAF. Methodology was taken care by SC, DF, MR‐S, and JAF. Software was taken care by AM, AB, AMNS, and JAF. Validation was taken care by LPA and LLS. Investigation was taken care by SC, DF, MR‐S, AB, AM, EF, BS, MG, and PL. Resources was taken care by AB, LLS, AMNS, and JAF. Data curation was taken care by SC, DF, MRS, AM, LPA, and JAF. Writing—original draft was taken care by SC, DF, and JAF. Writing—review and editing was taken care by SC, DF, LLS, and AMNS. Visualization was taken care by SC, DF, MRS, and JAF. Supervision and project administration were taken care by JAF. Funding acquisition was taken care by LLS and JAF.

### Peer review

The peer review history for this article is available at https://www.webofscience.com/api/gateway/wos/peer‐review/10.1002/1878‐0261.13733.

## Supporting information


**Fig. S1.** SLeA and sLeX are abundantly expressed in primary tumors and to a high extent in lymph node and distant metastases.
**Fig. S2.** Flow cytometric gate strategy to identify sLeA and sLeX‐positive leukocytes in peripheral blood of healthy donors.
**Fig. S3.** Expression of glycosyltransferases involved in sLe biosynthesis in CRC and normal adjacent tissues.
**Fig. S4.** Overexpression of highly correlated *FUT3*, *FUT4*, and *FUT6* associate with a favorable prognosis in CRC, whereas *FUT9* presents a trend link to worst prognosis.
**Fig. S5.** *ST3GAL3* is overexpressed in SLeA and SLeX‐positive compared with ‐negative tumors.
**Fig. S6.** Typical MS spectra reflecting the N‐glycome of metastatic colorectal tumor tissues used in this study.
**Fig. S7.** MS/MS spectra for unglycosylated peptides (top panels) and glycopeptides (bottom panels) supporting the expression of neuroendocrine‐related glycoproteins NCAM1 and TENM4 potentially carrying sialylated Lewis antigens.
**Fig. S8.** CRC tumors pooled for E‐selectin enrichment and glycoproteomic analysis showed evidence of relevant neovascularization (CD34+), neuroendocrine features (Synaptophysin+), and high levels of sialylated Lewis antigens in the same tissue area.
**Fig. S9.** Venn diagram highlighting E‐selectin affinity glycoproteins identified to date in the context of CRC.
**Fig. S10.** The SCTR receptor is highly expressed in colorectal tumors and may also be found in lymph node and distant metastases.
**Fig. S11.** *SCTR* and *SCT* overexpressions associate with decreased survival in advanced stage CRC.
**Fig. S12.** SCTR presents relevant expression in several human healthy organs (respiratory system; gastrointestinal tract; pancreas; kidney and urinary bladder).
**Fig. S13.** Flow cytometric gate strategy to identify sLeA and SCTR‐positive leukocytes in peripheral blood of healthy donors.

**Table S1.** Clinicopathological data of patients included in this study (*n* = 35).
**Table S2.** Clinicopathological characteristics of the CRC patients in the TCGA: COADREAD dataset used in this study.
**Table S3.** Flow cytometry to characterize the expression of sLeA and sLeX in peripheral blood of three healthy donors.
**Table S4.** N‐glycome of metastatic CRC tumor samples.
**Table S5.** O‐glycome of metastatic CRC tumor samples.
**Table S6.** CRC O‐glycoproteome analyzed in FFPE tumor tissues.
**Table S7.** CRC N‐glycoproteome analyzed in FFPE tumor tissues.
**Table S8.** List of proteins selected for target score.
**Table S9.** Flow cytometry to characterize the expression of sLeA and SCTR in peripheral blood of three healthy donors.

## Data Availability

Proteomics data generated during this study are included in Tables S6 and S7. The corresponding mass spectrometry data have been deposited to the ProteomeXchange Consortium via the PRIDE [1] partner repository with the dataset identifier PXD054727.

## References

[1] Morgan E , Arnold M , Gini A , Lorenzoni V , Cabasag CJ , Laversanne M , et al. Global burden of colorectal cancer in 2020 and 2040: incidence and mortality estimates from GLOBOCAN. Gut. 2023;72(2):338–344. 36604116 10.1136/gutjnl-2022-327736

[2] Molinari C , Marisi G , Passardi A , Matteucci L , de Maio G , Ulivi P . Heterogeneity in colorectal cancer: a challenge for personalized medicine? Int J Mol Sci. 2018;19(12):3733. 30477151 10.3390/ijms19123733 PMC6321493

[3] Fernandes E , Sores J , Cotton S , Peixoto A , Ferreira D , Freitas R , et al. Esophageal, gastric and colorectal cancers: looking beyond classical serological biomarkers towards glycoproteomics‐assisted precision oncology. Theranostics. 2020;10(11):4903–4928. 32308758 10.7150/thno.42480 PMC7163443

[4] Moran AB , Elgood‐Hunt G , van der Burgt YEM , Wuhrer M , Mesker WE , Tollenaar RAEM , et al. Serum N‐glycosylation RPLC‐FD‐MS assay to assess colorectal cancer surgical interventions. Biomolecules. 2023;13(6):896. 37371476 10.3390/biom13060896 PMC10295937

[5] Madunic K , Mayboroda OA , Zhang T , Weber J , Boons GJ , Morreau H , et al. Specific (sialyl‐)Lewis core 2 O‐glycans differentiate colorectal cancer from healthy colon epithelium. Theranostics. 2022;12(10):4498–4512. 35832079 10.7150/thno.72818 PMC9254241

[6] Boyaval F , van Zeijl R , Dalebout H , Holst S , van Pelt G , Fariña‐Sarasqueta A , et al. N‐glycomic signature of stage II colorectal cancer and its association with the tumor microenvironment. Mol Cell Proteomics. 2021;20:100057. 33581319 10.1074/mcp.RA120.002215 PMC7973300

[7] Popat S , Hubner R , Houlston RS . Systematic review of microsatellite instability and colorectal cancer prognosis. J Clin Oncol. 2005;23(3):609–618. 15659508 10.1200/JCO.2005.01.086

[8] Cotton S , Ferreira D , Soares J , Peixoto A , Relvas‐Santos M , Azevedo R , et al. Target score‐a proteomics data selection tool applied to esophageal cancer identifies GLUT1‐sialyl Tn glycoforms as biomarkers of cancer aggressiveness. Int J Mol Sci. 2021;22(4):1664. 33562270 10.3390/ijms22041664 PMC7915893

[9] Fernandes E , Freitas R , Ferreira D , Soares J , Azevedo R , Gaiteiro C , et al. Nucleolin‐Sle a glycoforms as E‐selectin ligands and potentially targetable biomarkers at the cell surface of gastric cancer cells. Cancers (Basel). 2020;12(4):861. 32252346 10.3390/cancers12040861 PMC7226152

[10] Peixoto A , Ferreira D , Azevedo R , Freitas R , Fernandes E , Relvas‐Santos M , et al. Glycoproteomics identifies HOMER3 as a potentially targetable biomarker triggered by hypoxia and glucose deprivation in bladder cancer. J Exp Clin Cancer Res. 2021;40(1):191. 34108014 10.1186/s13046-021-01988-6 PMC8188679

[11] Zhang T , Madunić K , Holst S , Zhang J , Jin C , ten Dijke P , et al. Development of a 96‐well plate sample preparation method for integrated N‐ and O‐glycomics using porous graphitized carbon liquid chromatography‐mass spectrometry. Mol Omics. 2020;16(4):355–363. 32281997 10.1039/c9mo00180h

[12] Peixoto A , Ferreira D , Miranda A , Relvas‐Santos M , Freitas R , Veth TS , et al. Multilevel plasticity and altered glycosylation drive aggressiveness in hypoxic and glucose‐deprived bladder cancer cells. bioRxiv. 2024. 10.1101/2023.10.21.561355 PMC11791300 39906564

[13] Ceroni A , Maass K , Geyer H , Geyer R , Dell A , Haslam SM . GlycoWorkbench: a tool for the computer‐assisted annotation of mass spectra of glycans. J Proteome Res. 2008;7(4):1650–1659. 18311910 10.1021/pr7008252

[14] Soares J , Eiras M , Ferreira D , Santos DAR , Relvas‐Santos M , Santos B , et al. Stool glycoproteomics signatures of pre‐cancerous lesions and colorectal cancer. Int J Mol Sci. 2024;25(7):3722. 38612533 10.3390/ijms25073722 PMC11012158

[15] Freitas R , Miranda A , Ferreira D , Relvas‐Santos M , Castro F , Ferreira E , et al. A multivalent CD44 glycoconjugate vaccine candidate for cancer immunotherapy. J Control Release. 2024;367:540–556. 38301927 10.1016/j.jconrel.2024.01.065

[16] Carrascal MA , Silva M , Ferreira JA , Azevedo R , Ferreira D , Silva AMN , et al. A functional glycoproteomics approach identifies CD13 as a novel E‐selectin ligand in breast cancer. Biochim Biophys Acta Gen Subj. 2018;1862(9):2069–2080. 29777742 10.1016/j.bbagen.2018.05.013

[17] Shannon P , Markiel A , Ozier O , Baliga NS , Wang JT , Ramage D , et al. Cytoscape: a software environment for integrated models of biomolecular interaction networks. Genome Res. 2003;13(11):2498–2504. 14597658 10.1101/gr.1239303 PMC403769

[18] Bindea G , Mlecnik B , Hackl H , Charoentong P , Tosolini M , Kirilovsky A , et al. ClueGO: a Cytoscape plug‐in to decipher functionally grouped gene ontology and pathway annotation networks. Bioinformatics. 2009;25(8):1091–1093. 19237447 10.1093/bioinformatics/btp101 PMC2666812

[19] Bindea G , Galon J , Mlecnik B . CluePedia Cytoscape plugin: pathway insights using integrated experimental and in silico data. Bioinformatics. 2013;29(5):661–663. 23325622 10.1093/bioinformatics/btt019 PMC3582273

[20] Uhlen M , Fagerberg L , Hallström BM , Lindskog C , Oksvold P , Mardinoglu A , et al. Proteomics. Tissue‐based map of the human proteome. Science. 2015;347(6220):1260419. 25613900 10.1126/science.1260419

[21] Livak KJ , Schmittgen TD . Analysis of relative gene expression data using real‐time quantitative PCR and the 2(‐Delta Delta C(T)) method. Methods. 2001;25(4):402–408. 11846609 10.1006/meth.2001.1262

[22] Aviles EC , Goodrich LV . Configuring a robust nervous system with fat cadherins. Semin Cell Dev Biol. 2017;69:91–101. 28603077 10.1016/j.semcdb.2017.06.001 PMC5582996

[23] Rambaldi B , Kim HT , Arihara Y , Asano T , Reynolds C , Manter M , et al. Phenotypic and functional characterization of the CD6‐ALCAM T‐cell co‐stimulatory pathway after allogeneic cell transplantation. Haematologica. 2022;107(11):2617–2629. 35484649 10.3324/haematol.2021.280444 PMC9614543

[24] Slattery ML , Lundgreen A , Wolff RK . MAP kinase genes and colon and rectal cancer. Carcinogenesis. 2012;33(12):2398–2408. 23027623 10.1093/carcin/bgs305 PMC3510742

[25] Zhou G , Yang J , Song P . Correlation of ERK/MAPK signaling pathway with proliferation and apoptosis of colon cancer cells. Oncol Lett. 2019;17(2):2266–2270. 30675292 10.3892/ol.2018.9857 PMC6341783

[26] Deschepper FM , Zoppi R , Pirro M , Hensbergen PJ , Dall'Olio F , Kotsias M , et al. L1CAM as an E‐selectin ligand in colon cancer. Int J Mol Sci. 2020;21(21):8286. 33167483 10.3390/ijms21218286 PMC7672641

[27] Suzuki N , Numakawa T , Chou J , Vega S , Mizuniwa C , Sekimoto K , et al. Teneurin‐4 promotes cellular protrusion formation and neurite outgrowth through focal adhesion kinase signaling. FASEB J. 2014;28(3):1386–1397. 24344332 10.1096/fj.13-241034 PMC3929675

[28] Bai JJ , Tan CD , Chow BKC . Secretin, at the hub of water‐salt homeostasis. Am J Physiol Renal Physiol. 2017;312(5):F852–F860. 27279485 10.1152/ajprenal.00191.2015

[29] Afroze S , Meng F , Jensen K , McDaniel K , Rahal K , Onori P , et al. The physiological roles of secretin and its receptor. Ann Transl Med. 2013;1(3):29. 25332973 10.3978/j.issn.2305-5839.2012.12.01 PMC4200670

[30] Ferreira JA , Magalhães A , Gomes J , Peixoto A , Gaiteiro C , Fernandes E , et al. Protein glycosylation in gastric and colorectal cancers: toward cancer detection and targeted therapeutics. Cancer Lett. 2017;387:32–45. 26828132 10.1016/j.canlet.2016.01.044

[31] Holst S , Wuhrer M , Rombouts Y . Glycosylation characteristics of colorectal cancer. Adv Cancer Res. 2015;126:203–256. 25727149 10.1016/bs.acr.2014.11.004

[32] Tomlinson J , Wang JL , Barsky SH , Lee MC , Bischoff J , Nguyen M . Human colon cancer cells express multiple glycoprotein ligands for E‐selectin. Int J Oncol. 2000;16(2):347–353. 10639580 10.3892/ijo.16.2.347

[33] Kobayashi K , Matsumoto S , Morishima T , Kawabe T , Okamoto T . Cimetidine inhibits cancer cell adhesion to endothelial cells and prevents metastasis by blocking E‐selectin expression. Cancer Res. 2000;60(14):3978–3984. 10919677

[34] Kohler S , Ullrich S , Richter U , Schumacher U . E‐/P‐selectins and colon carcinoma metastasis: first in vivo evidence for their crucial role in a clinically relevant model of spontaneous metastasis formation in the lung. Br J Cancer. 2010;102(3):602–609. 20010946 10.1038/sj.bjc.6605492 PMC2822933

[35] Sackstein R . The lymphocyte homing receptors: gatekeepers of the multistep paradigm. Curr Opin Hematol. 2005;12(6):444–450. 16217160 10.1097/01.moh.0000177827.78280.79

[36] Hidalgo A , Peired AJ , Wild MK , Vestweber D , Frenette PS . Complete identification of E‐selectin ligands on neutrophils reveals distinct functions of PSGL‐1, ESL‐1, and CD44. Immunity. 2007;26(4):477–489. 17442598 10.1016/j.immuni.2007.03.011 PMC4080624

[37] Burdick MM , Chu JT , Godar S , Sackstein R . HCELL is the major E‐ and L‐selectin ligand expressed on LS174T colon carcinoma cells. J Biol Chem. 2006;281(20):13899–13905. 16565092 10.1074/jbc.M513617200

[38] Thomas SN , Zhu F , Schnaar RL , Alves CS , Konstantopoulos K . Carcinoembryonic antigen and CD44 variant isoforms cooperate to mediate colon carcinoma cell adhesion to E‐ and L‐selectin in shear flow. J Biol Chem. 2008;283(23):15647–15655. 18375392 10.1074/jbc.M800543200 PMC2414264

[39] Larsson A , Johansson ME , Wangefjord S , Gaber A , Nodin B , Kucharzewska P , et al. Overexpression of podocalyxin‐like protein is an independent factor of poor prognosis in colorectal cancer. Br J Cancer. 2011;105(5):666–672. 21829192 10.1038/bjc.2011.295 PMC3188928

[40] Boyaval F , Dalebout H , van Zeijl R , Wang W , Fariña‐Sarasqueta A , Lageveen‐Kammeijer GSM , et al. High‐mannose N‐glycans as malignant progression markers in early‐stage colorectal cancer. Cancers (Basel). 2022;14(6):1552. 35326703 10.3390/cancers14061552 PMC8945895

[41] Carrascal MA , Silva M , Ramalho JS , Pen C , Martins M , Pascoal C , et al. Inhibition of fucosylation in human invasive ductal carcinoma reduces E‐selectin ligand expression, cell proliferation, and ERK1/2 and p38 MAPK activation. Mol Oncol. 2018;12(5):579–593. 29215790 10.1002/1878-0261.12163 PMC5928367

[42] Colomb F , Krzewinski‐Recchi MA , Steenackers A , Vincent A , Harduin‐Lepers A , Delannoy P , et al. TNF up‐regulates ST3GAL4 and sialyl‐Lewisx expression in lung epithelial cells through an intronic ATF2‐responsive element. Biochem J. 2017;474(1):65–78. 27821620 10.1042/BCJ20160602

[43] Breen KC , Coughlan CM , Hayes FD . The role of glycoproteins in neural development function, and disease. Mol Neurobiol. 1998;16(2):163–220. 9588627 10.1007/BF02740643

[44] Schultheis M , Diestel S , Schmitz B . The role of cytoplasmic serine residues of the cell adhesion molecule L1 in neurite outgrowth, endocytosis, and cell migration. Cell Mol Neurobiol. 2007;27(1):11–31. 17151951 10.1007/s10571-006-9113-1 PMC11517402

[45] Yamasaki M , Thompson P , Lemmon V . CRASH syndrome: mutations in L1CAM correlate with severity of the disease. Neuropediatrics. 1997;28(3):175–178. 9266556 10.1055/s-2007-973696 PMC1563987

[46] Bateman A , Jouet M , MacFarlane J , Du JS , Kenwrick S , Chothia C . Outline structure of the human L1 cell adhesion molecule and the sites where mutations cause neurological disorders. EMBO J. 1996;15(22):6050–6059. 8947027 PMC452426

[47] Chen DS , Mellman I . Elements of cancer immunity and the cancer‐immune set point. Nature. 2017;541(7637):321–330. 28102259 10.1038/nature21349

[48] He X , Xu C . Immune checkpoint signaling and cancer immunotherapy. Cell Res. 2020;30(8):660–669. 32467592 10.1038/s41422-020-0343-4 PMC7395714

[49] Liu K , Tan S , Jin W , Guan J , Wang Q , Sun H , et al. N‐glycosylation of PD‐1 promotes binding of camrelizumab. EMBO Rep. 2020;21(12):e51444. 33063473 10.15252/embr.202051444 PMC7726772

[50] Lu D , Xu Z , Zhang D , Jiang M , Liu K , He J , et al. PD‐1 N58‐glycosylation‐dependent binding of monoclonal antibody cemiplimab for immune checkpoint therapy. Front Immunol. 2022;13:826045. 35309324 10.3389/fimmu.2022.826045 PMC8924070

[51] Silva M , Martin KC , Mondal N , Sackstein R . sLeX expression delineates distinct functional subsets of human blood central and effector memory T cells. J Immunol. 2020;205(7):1920–1932. 32868410 10.4049/jimmunol.1900679 PMC10636707

[52] Silva M , Videira PA , Sackstein R . E‐selectin ligands in the human mononuclear phagocyte system: implications for infection, inflammation, and immunotherapy. Front Immunol. 2017;8:1878. 29403469 10.3389/fimmu.2017.01878 PMC5780348

[53] Cohen EN , Fouad TM , Lee BN , Arun BK , Liu D , Tin S , et al. Elevated serum levels of sialyl Lewis X (sLe(X)) and inflammatory mediators in patients with breast cancer. Breast Cancer Res Treat. 2019;176(3):545–556. 31054033 10.1007/s10549-019-05258-0 PMC6653624

[54] Trinchera M , Aronica A , Dall'Olio F . Selectin ligands sialyl‐Lewis a and sialyl‐Lewis x in gastrointestinal cancers. Biology (Basel). 2017;6(1):16. 28241499 10.3390/biology6010016 PMC5372009

[55] Magalhaes A , Marcos‐Pinto R , Nairn AV , Dela Rosa M , Ferreira RM , Junqueira‐Neto S , et al. *Helicobacter pylori* chronic infection and mucosal inflammation switches the human gastric glycosylation pathways. Biochim Biophys Acta. 2015;1852(9):1928–1939. 26144047 10.1016/j.bbadis.2015.07.001 PMC4638172

[56] Hirakawa M , Takimoto R , Tamura F , Yoshida M , Ono M , Murase K , et al. Fucosylated TGF‐beta receptors transduces a signal for epithelial‐mesenchymal transition in colorectal cancer cells. Br J Cancer. 2014;110(1):156–163. 24253505 10.1038/bjc.2013.699 PMC3887298

[57] Geiger T . Tackling tumor complexity with single‐cell proteomics. Nat Methods. 2023;20(3):324–326. 36899159 10.1038/s41592-023-01784-4

[58] De Vargas Roditi L , Jacobs A , Rueschoff JH , Bankhead P , Chevrier S , Jackson HW , et al. Single‐cell proteomics defines the cellular heterogeneity of localized prostate cancer. Cell Rep Med. 2022;3(4):100604. 35492239 10.1016/j.xcrm.2022.100604 PMC9044103

[59] Li M , Zuo J , Yang K , Wang P , Zhou S . Proteomics mining of cancer hallmarks on a single‐cell resolution. Mass Spectrom Rev. 2023;43:1019–1040. 37051664 10.1002/mas.21842

[60] Drokhlyansky E , Smillie CS , van Wittenberghe N , Ericsson M , Griffin GK , Eraslan G , et al. The human and mouse enteric nervous system at single‐cell resolution. Cell. 2020;182(6):1606–1622.e23. 32888429 10.1016/j.cell.2020.08.003 PMC8358727

[61] Klussmeier A , Aurich S , Niederstadt L , Wiedenmann B , Grötzinger C . Secretin receptor as a target in gastrointestinal cancer: expression analysis and ligand development. Biomedicine. 2022;10(3):536. 10.3390/biomedicines10030536 PMC8944975 35327338

[62] Lee M , Waser B , Reubi JC , Pellegata NS . Secretin receptor promotes the proliferation of endocrine tumor cells via the PI3K/AKT pathway. Mol Endocrinol. 2012;26(8):1394–1405. 22692904 10.1210/me.2012-1055 PMC5416982

[63] Buchsbaum DJ . Experimental tumor targeting with radiolabeled ligands. Cancer. 1997;80(12 Suppl):2371–2377. 9406685 10.1002/(sici)1097-0142(19971215)80:12+<2371::aid-cncr6>3.3.co;2-a

[64] Fernandes E , Peixoto A , Neves M , Afonso LP , Santos LL , Ferreira JA . Humoral response against sialyl‐Le(a) glycosylated protein species in esophageal cancer: insights for immunoproteomic studies. Electrophoresis. 2015;36(23):2902–2907. 26333169 10.1002/elps.201500270

[65] Itai S , Nishikata J , Yoneda T , Ohmori K , Yamabe H , Arii S , et al. Tissue distribution of 2‐3 and 2‐6 sialyl Lewis A antigens and significance of the ratio of two antigens for the differential diagnosis of malignant and benign disorders of the digestive tract. Cancer. 1991;67(6):1576–1587. 2001547 10.1002/1097-0142(19910315)67:6<1576::aid-cncr2820670620>3.0.co;2-2

[66] Li X , Peng X , Yang S , Wei S , Fan Q , Liu J , et al. Targeting tumor innervation: premises, promises, and challenges. Cell Death Dis. 2022;8(1):131. 10.1038/s41420-022-00930-9 PMC8956600 35338118

[67] Gysler SM , Drapkin R . Tumor innervation: peripheral nerves take control of the tumor microenvironment. J Clin Invest. 2021;131(11):e147276. 34060481 10.1172/JCI147276 PMC8159682

[68] Cervantes‐Villagrana RD , Albores‐García D , Cervantes‐Villagrana AR , García‐Acevez SJ . Tumor‐induced neurogenesis and immune evasion as targets of innovative anti‐cancer therapies. Signal Transduct Target Ther. 2020;5(1):99. 32555170 10.1038/s41392-020-0205-z PMC7303203

[69] Ferreira IG , Carrascal M , Mineiro AG , Bugalho A , Borralho P , Silva Z , et al. Carcinoembryonic antigen is a sialyl Lewis x/a carrier and an E‐selectin ligand in non‐small cell lung cancer. Int J Oncol. 2019;55(5):1033–1048. 31793656 10.3892/ijo.2019.4886 PMC6776192

[70] Silverman DA , Martinez VK , Dougherty PM , Myers JN , Calin GA , Amit M . Cancer‐associated neurogenesis and nerve‐cancer cross‐talk. Cancer Res. 2021;81(6):1431–1440. 33334813 10.1158/0008-5472.CAN-20-2793 PMC7969424

