# Supplementary material for: E‐selectin affinity glycoproteomics reveals neuroendocrine proteins and the secretin receptor as a poor‐prognosis signature in colorectal cancer
Source: Mol Oncol. 2024 Nov 7;19(3):635–58. doi: 10.1002/1878-0261.13733 (PMC11887675; doi:10.1002/1878-0261.13733)
Supplement: Supplementary file 1 — Fig. S1. SLeA and sLeX are abundantly expressed in primary tumors and to a high extent in lymph node and distant metastases. Fig. S2. Flow cytometric gate strategy to identify sLeA and sLeX‐positive leukocytes in peripheral blood of healthy donors. Fig. S3. Expression of glycosyltransferases involved in sLe biosynthesis in CRC and normal adjacent tissues. Fig. S4. Overexpression of highly correlated FUT3, FUT4, and FUT6 associate with a favorable prognosis in CRC, whereas FUT9 presents a trend link to worst prognosis. Fig. S5. ST3GAL3 is overexpressed in SLeA and SLeX‐positive compared with ‐negative tumors. Fig. S6. Typical MS spectra reflecting the N‐glycome of metastatic colorectal tumor tissues used in this study. Fig. S7. MS/MS spectra for unglycosylated peptides (top panels) and glycopeptides (bottom panels) supporting the expression of neuroendocrine‐related glycoproteins NCAM1 and TENM4 potentially carrying sialylated Lewis antigens. Fig. S8. CRC tumors pooled for E‐selectin enrichment and glycoproteomic analysis showed evidence of relevant neovascularization (CD34+), neuroendocrine features (Synaptophysin+), and high levels of sialylated Lewis antigens in the same tissue area. Fig. S9. Venn diagram highlighting E‐selectin affinity glycoproteins identified to date in the context of CRC. Fig. S10. The SCTR receptor is highly expressed in colorectal tumors and may also be found in lymph node and distant metastases. Fig. S11. SCTR and SCT overexpressions associate with decreased survival in advanced stage CRC. Fig. S12. SCTR presents relevant expression in several human healthy organs (respiratory system; gastrointestinal tract; pancreas; kidney and urinary bladder). Fig. S13. Flow cytometric gate strategy to identify sLeA and SCTR‐positive leukocytes in peripheral blood of healthy donors. [file MOL2-19-635-s002.doc]

**E-selectin Affinity Glycoproteomics Reveals Neuroendocrine Proteins and the Secretin Receptor as a Poor Prognosis Signature in Colorectal Cancer**

**Supporting Figures**

Sofia Cotton^1,2^, Dylan Ferreira^1,2,3^, Marta Relvas-Santos^1,2,3,4^, Andreia Brandão^1^, Luís Pedro Afonso^1,5^, Andreia Miranda^1,2,3^, Eduardo Ferreira^1^, Beatriz Santos^1,2^, Martina Gonçalves^1,2^, Lúcio Lara Santos^1,6,7^, André M N Silva^2,4^, José Alexandre Ferreira^1,2^

^1^Research Center of IPO-Porto (CI-IPOP) / RISE@CI-IPOP (Health Research Network), Portuguese Oncology Institute of Porto (IPO-Porto) / Porto Comprehensive Cancer Center (P.ccc) Raquel Seruca, Porto, Portugal; ^2^Institute of Biomedical Sciences Abel Salazar (ICBAS), University of Porto, Porto, Portugal; ^3^i3S – Instituto de Investigação e Inovação em Saúde, Universidade do Porto, Porto, Portugal; ^4^LAQV-REQUIMTE, Department of Chemistry and Biochemistry, Faculty of Sciences, University of Porto, Porto, Portugal; ^5^Pathology Department, Portuguese Oncology Institute of Porto, Porto, Portugal; ^6^Health School of University Fernando Pessoa, Porto, Portugal; ^7^Department of Surgical Oncology, Portuguese Oncology Institute of Porto, Porto, Portugal.

**Corresponding author**

José Alexandre Ferreira

Experimental Pathology and Therapeutics Group,

Research Centre, Portuguese Oncology Institute of Porto,

R. Dr. António Bernardino de Almeida 4200-072 Porto,

Portugal; Tel. +351 225084000 (ext. 5111).

Email: [jose.a.ferreira@ipoporto.min-saude.pt](mailto:jose.a.ferreira@ipoporto.min-saude.pt)


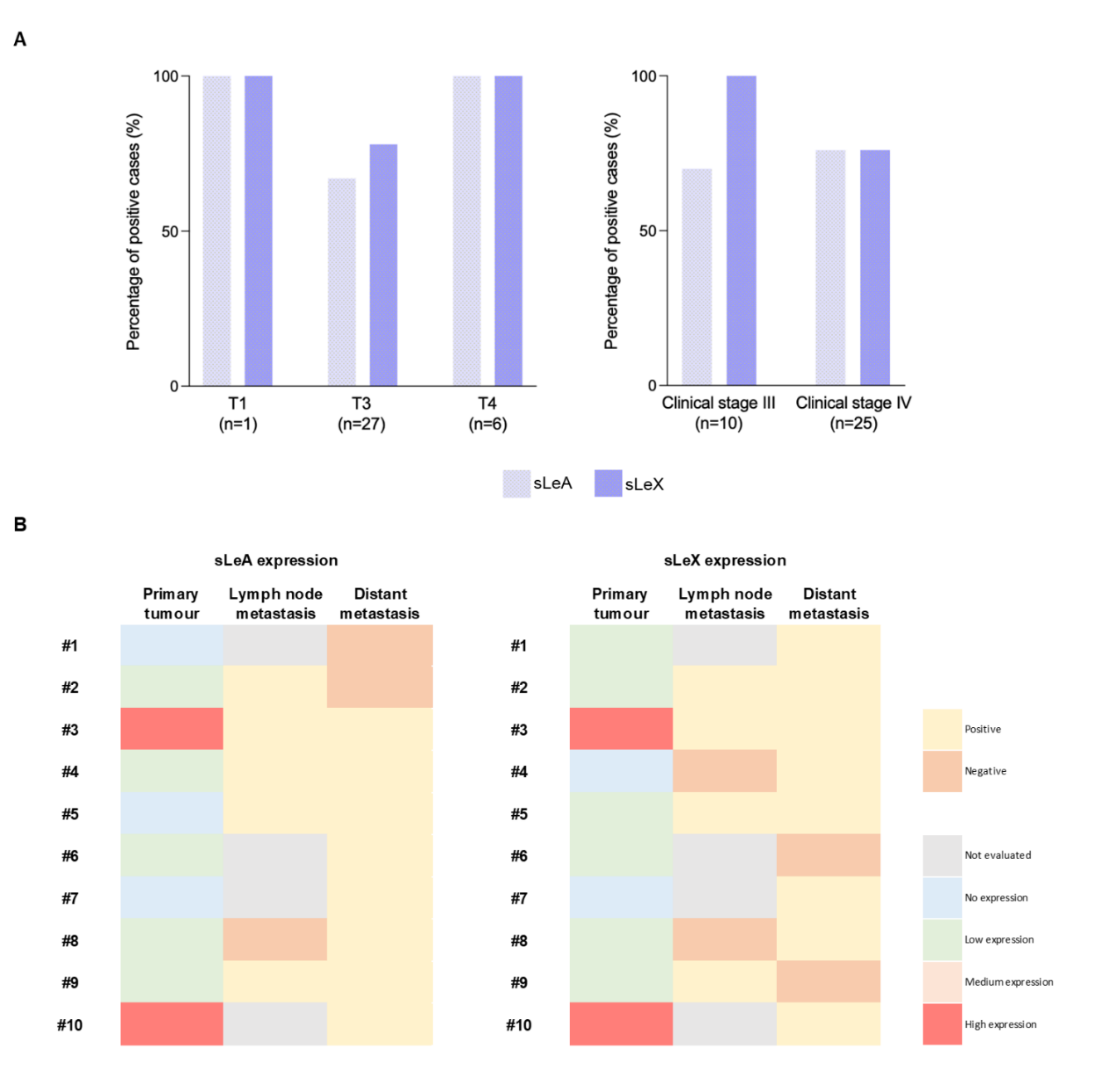


**Figure S1. SLeA and sLeX are abundantly expressed in primary tumours and to a high extent in lymph node and distant metastases. A) SLe glycoepitopes expression according to the degree of invasion (pT) and clinical staging.** A Chi-square analysis was performed and showed no associations of sLeA and sLeX with T (sLeA: *p*=0.25; sLeX: *p*=0.512) and clinical stages (sLeA: *p*=0.389; sLeX: *p*=0.109). Statistical significance was considered when p≤0.05. **B) SLe glycoepitopes expression in primary tumours vs lymph node and distant metastases.**


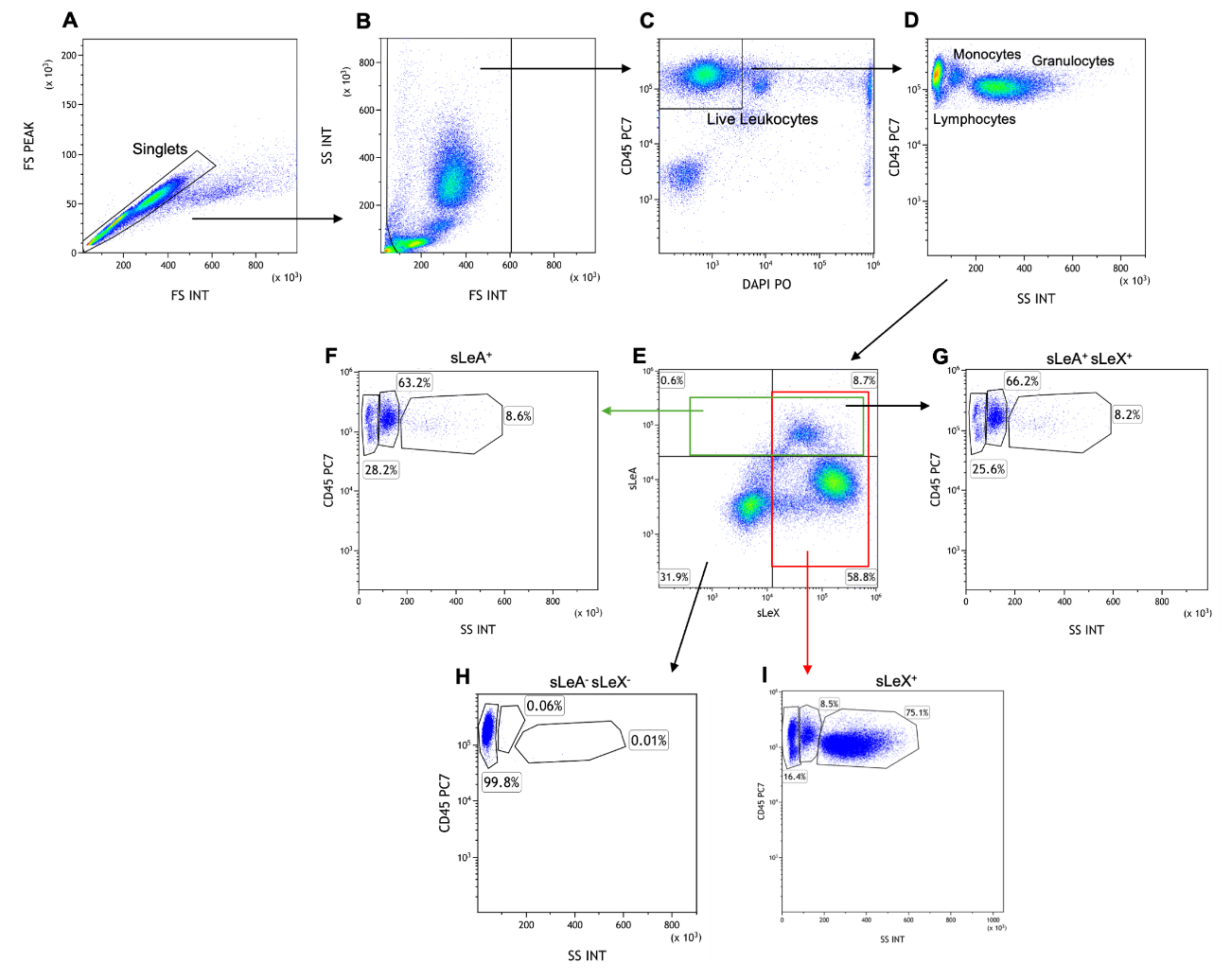


**Figure S2. Flow cytometric gate strategy to identify sLeA and sLeX positive leukocytes in peripheral blood of healthy donors.** **A)** Singlets are selected according to forward scatter-height (FSC-H) versus forward scatter-area (FSC-A). **B)** Leukocytes are selected, and erythrocytes and debris were excluded using side scatter-area (SSC-A) versus FSC-A. **C)** Live leukocytes are isolated by selecting the CD45 positive and DAPI negative cells. **D)** Live leukocytes are subtyped into lymphocytes, monocytes, and granulocytes according to CD45 expression and SSC-A dispersion. **E)** sLeA+/sLeX+ leukocytes are isolated using fluorochrome-coupled specific antibodies. **F)** Isolating the sLeA positive leukocytes, 30.0% of the cells are lymphocytes, 55.4% are monocytes, and 7.9% are granulocytes. **G)** Isolating the double positive leukocytes for sLeA and sLeX, 24.9% of the cells are lymphocytes, 60.0% are monocytes, and 7.9% are granulocytes. **H)** Isolating the double negative leukocytes for sLeA and sLeX, all the events are lymphocytes. **I)** Isolating the sLeX positive leukocytes, 13.6% of the cells are lymphocytes, 0.3% are monocytes, and 84.5% are granulocytes.

**
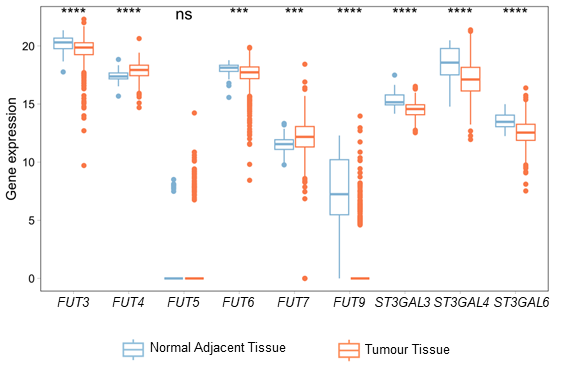
**

**Figure S3. Expression of glycosyltransferases involved in sLe biosynthesis in CRC and normal adjacent tissues.** The analysis of transcripts encoding key glycosyltransferases involved in sLe antigen biosynthesis was conducted on 598 tumour samples and 51 normal adjacent tissue samples from the The Cancer Genome Atlas (TCGA) database. *FUT4* and *FUT7* mostly linked to sLeX biosynthesis, were significantly overexpressed, whereas all the other glycosyltransferases were downregulated. *FUT5* is residually expressed by few cases in healthy tissues and CRC. Wilcoxon rank test was performed to assess differences between sample tissue types (normal adjacent tissues vs tumour tissue). Error bars extend to 1.5 times the interquartile range (IQR) from the box edges. Null hypothesis was rejected when p≤0.05.


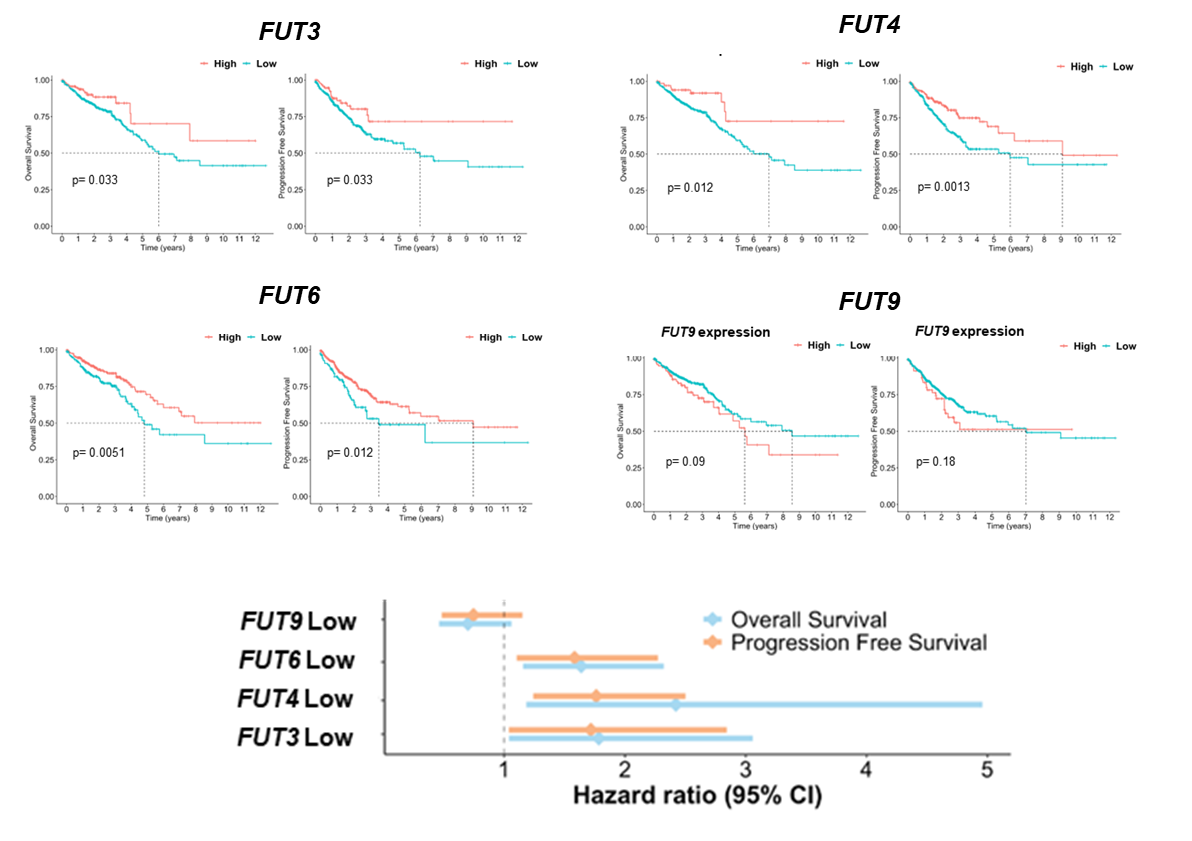


**Figure S4.** **Overexpression of highly correlated *FUT3*, *FUT4* and *FUT6* associate with a favorable prognosis in CRC, whereas *FUT9* presents a trend link to worst prognosis.** TCGA analysis revealed that high *FUT3*, *FUT4*, and *FUT6* transcripts associated with favourable overall and disease-free survivals. Also, decreased *FUT3*, *FUT4*, and *FUT6* correlated with higher cancer-related mortality and progression risk (FUT3low: HR-1.8, p=0.04/ HR-1.7, p=0.04; *FUT4*low: HR-2.4, p=0.02/ HR-1.8, p=0.002; FUT6low: HR=1.6, p=0.01/ HR-1.6, p=0.01). Notably, *FUT3* and *FUT6* showed a strong positive correlation. Conversely, *FUT9* overexpression, inversely correlated with *FUT3* and *FUT6*, trended with decreased overall survival. Additionally, tumors with low *FUT9* mRNA levels had lower hazard ratios for overall (HR: 0.7, p=0.09) and disease-free survivals (HR:0.7, p=0.18). The *p* value concerns log-rank test and Cox Proportional Hazards Regression Analysis. Statistical significance was determined at *p* ≤ 0.05. Statistical significance was determined at *p* ≤ 0.05.


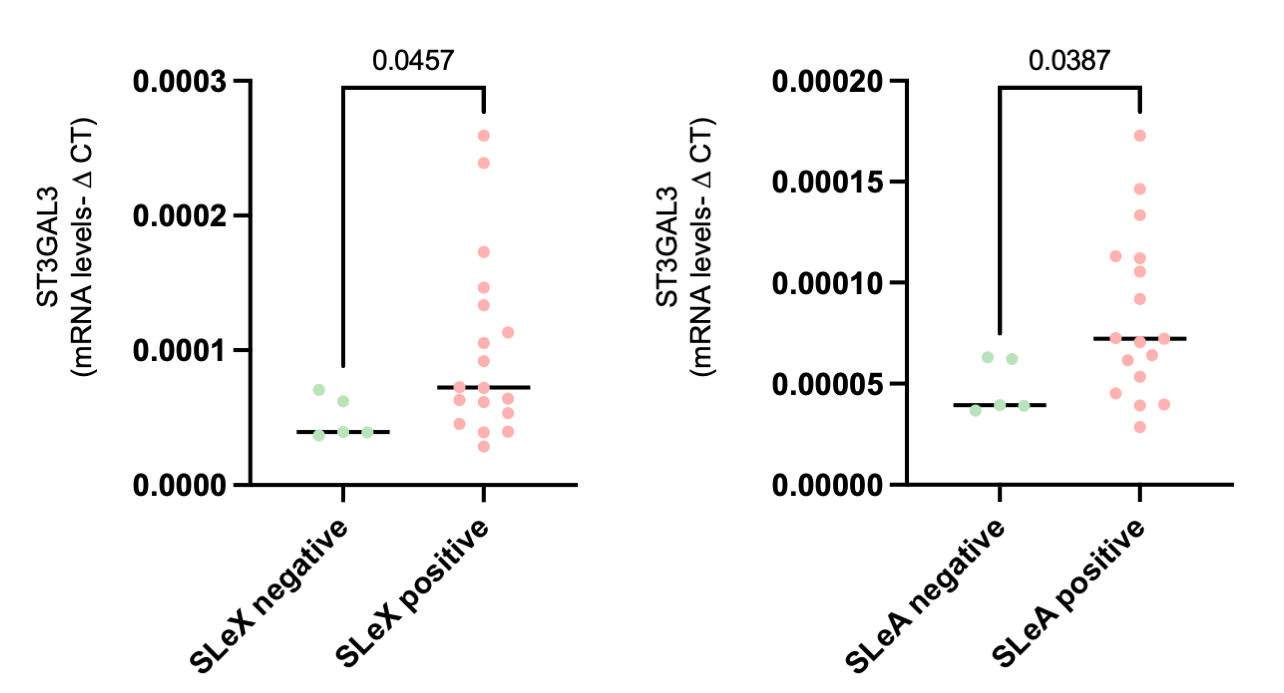


**Figure S5. *ST3GAL3* is overexpressed in sLeA and sLeX positive compared to negative tumours.** Number of evaluated tumours: sLeXnegative: 5; sLeXpositive: 18; sLeAnegative: 5; sLeApositive: 15. A Wilcoxon rank test was used to assess the differences between groups following the assessment of normality with the Shapiro-Wilk test. *P*-value of 0.05 or lower was considered statistically significant.


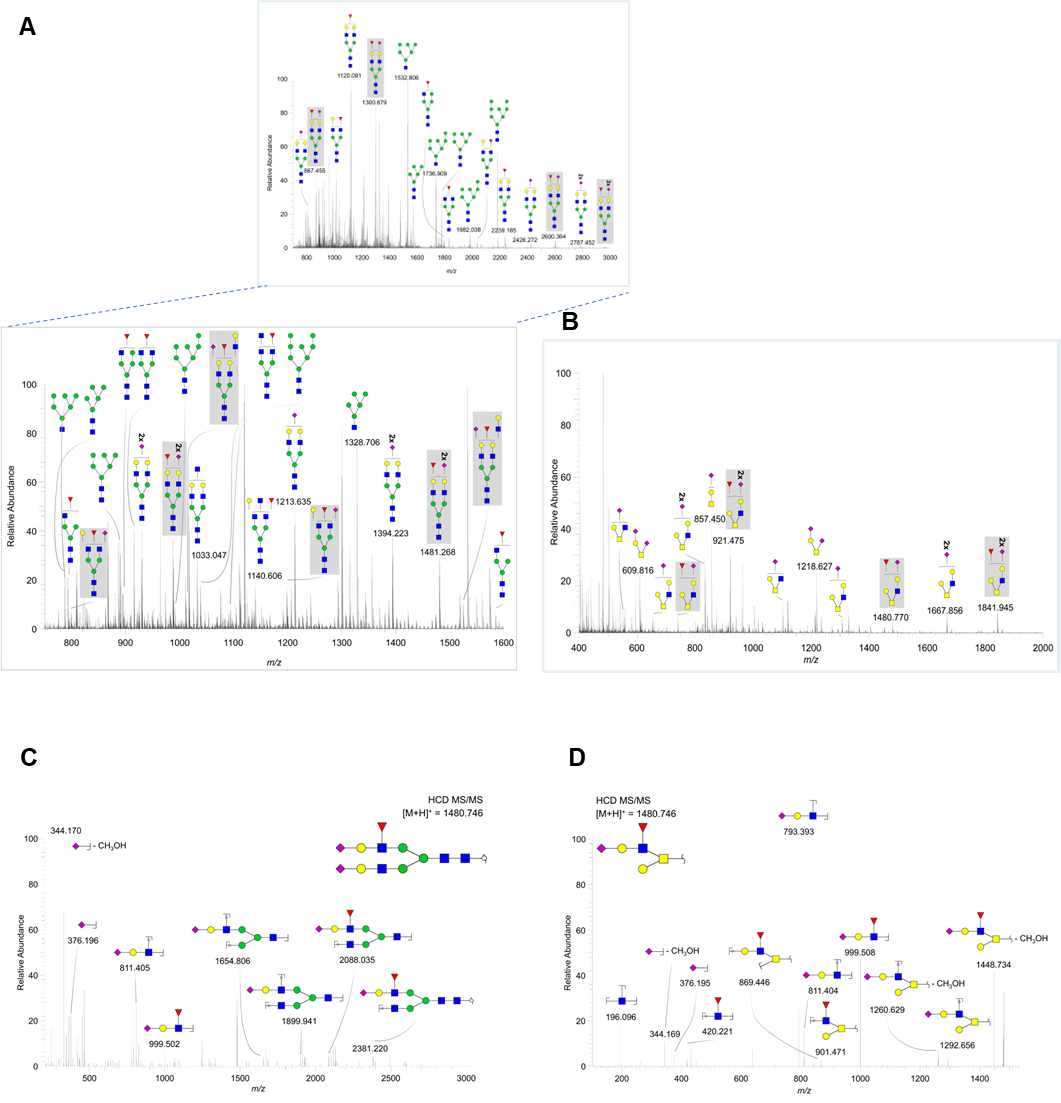


**Figure S6. A) Typical MS spectra reflecting the *N*-glycome of metastatic colorectal tumor tissues used in this study.** Dashed glycans are consistent with carrying sialylated Lewis antigens as terminal epitopes. **B) Typical MS spectra reflecting the *O*-glycome of metastatic stage IV colorectal tumor tissues used in this study.** **C) MS/MS of a complex *N*-glycan carrying sialylated Lewis antigens.** The spectrum highlights typical glycosidic linkage fragments consistent with the presence of sialylated Lewis antigens. **D) MS/MS of an extended core 2 *O*-glycan carrying sialylated Lewis antigens.**


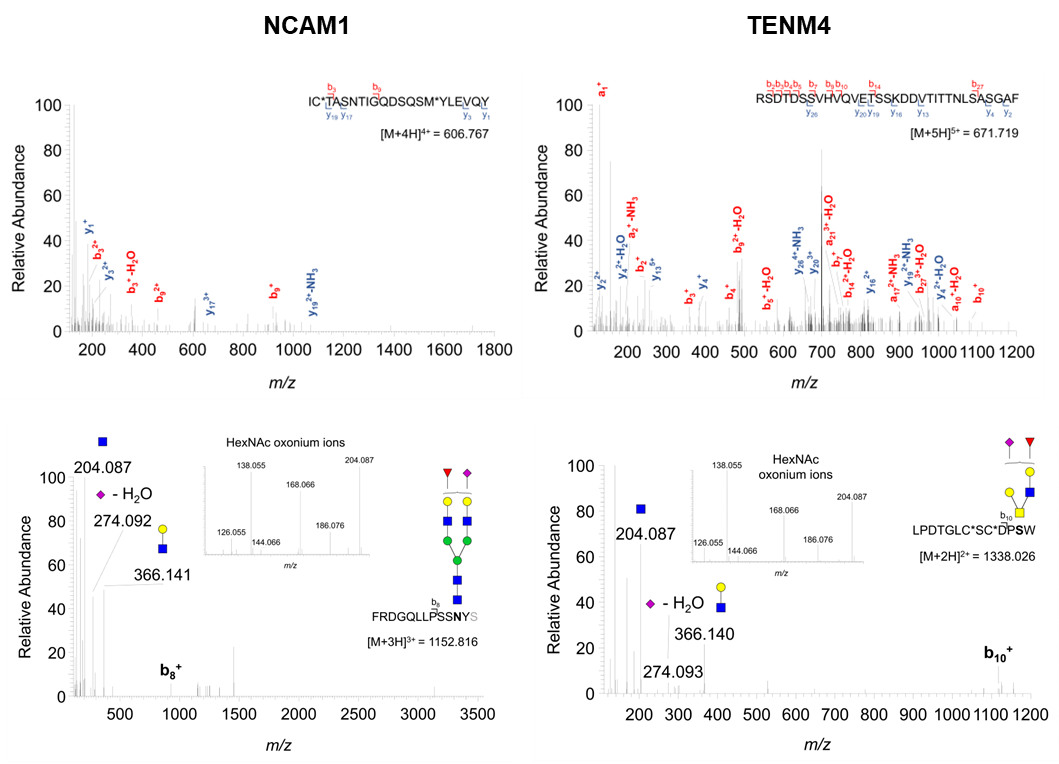


**Figure S7. MS/MS spectra for unglycosylated peptides (top panels) and glycopeptides (bottom panels) supporting the expression of neuroendocrine-related glycoproteins NCAM1 and TENM4 potentially carrying sialylated Lewis antigens.** * Modification of cysteine with carbamidomethyl

**
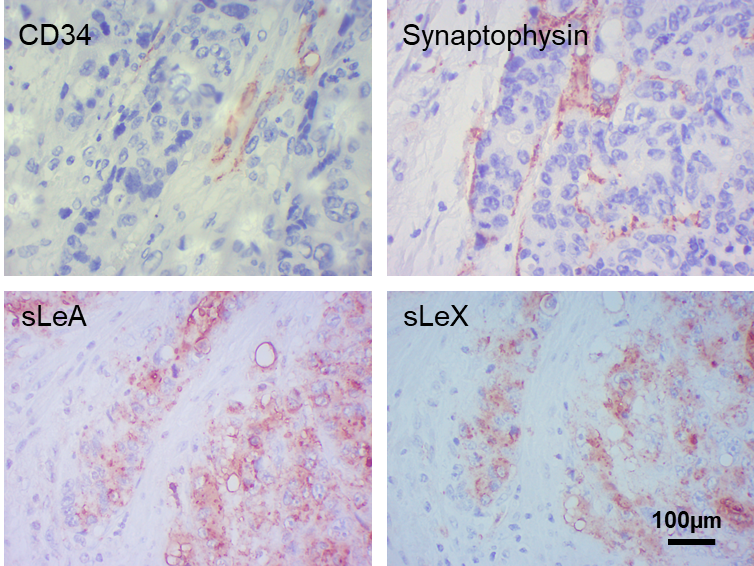
Figure S8. CRC tumours pooled for E-selectin enrichment and glycoproteomic analysis showed evidence of relevant neovascularization (CD34+), neuroendocrine features (Synaptophysin+), and high levels of sialylated Lewis antigens in the same tissue area.**

**
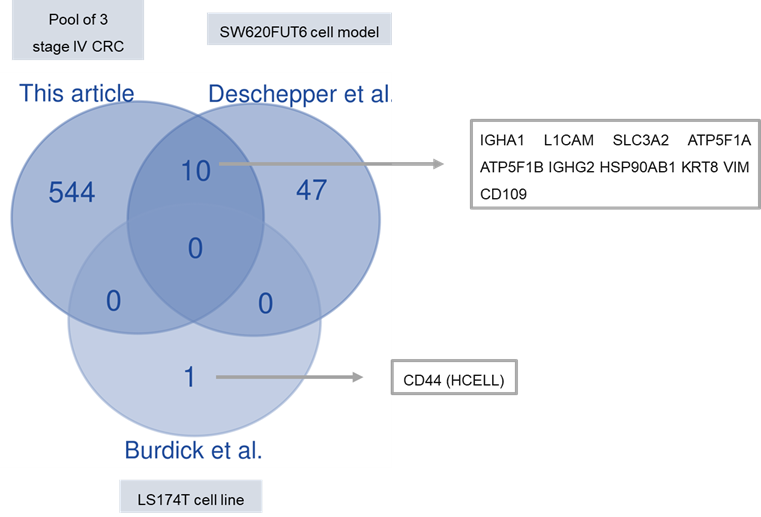
**

**Figure S9. Venn Diagram highlighting E-selectin affinity glycoproteins identified to date in the context of CRC.** The Venn Diagram highlights some common proteins between our study and the analysis of a sialyl-Lewis overexpressing cell line, including L1CAM which is a typical nervous system-associated glycoprotein.

**
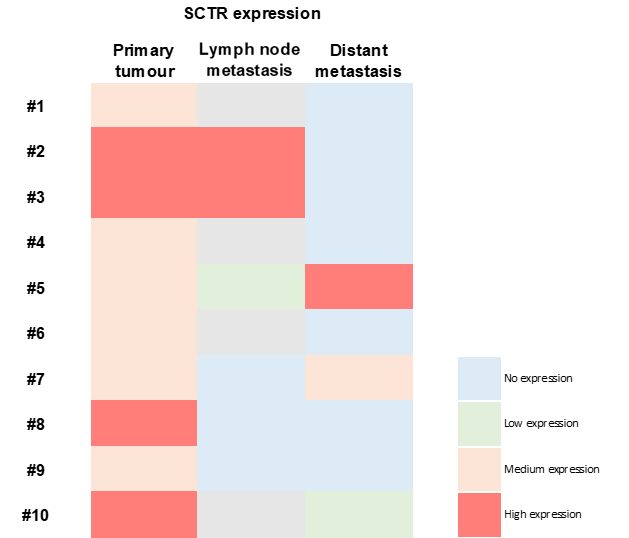
**

**Figure S10. The SCTR receptor is highly expressed in colorectal tumours and may also be found in lymph node and distant metastases.**

**
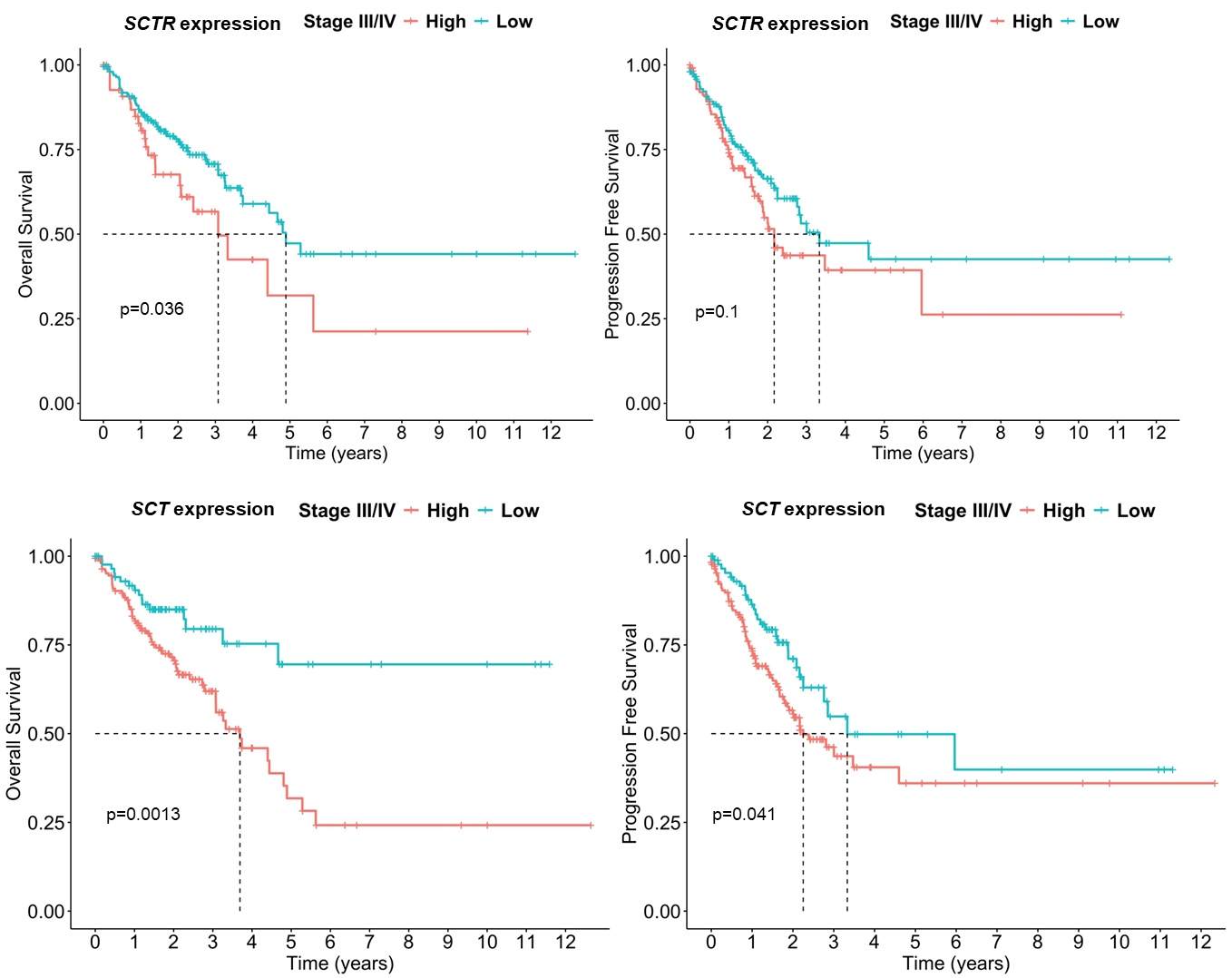
**

**Figure S11. *SCTR* and *SCT* overexpressions associate with decreased survival in advanced stage CRC.** *SCTR* overexpression associates with decreased overall survival in advanced stage (III/IV) CRC patients; whereas *SCT* overexpression associates with both overall and progression free survivals. The log-rank test and Cox Proportional Hazards Regression Analysis were employed to determine the *p* value. Statistical significance was considered when *p* ≤ 0.05.

**
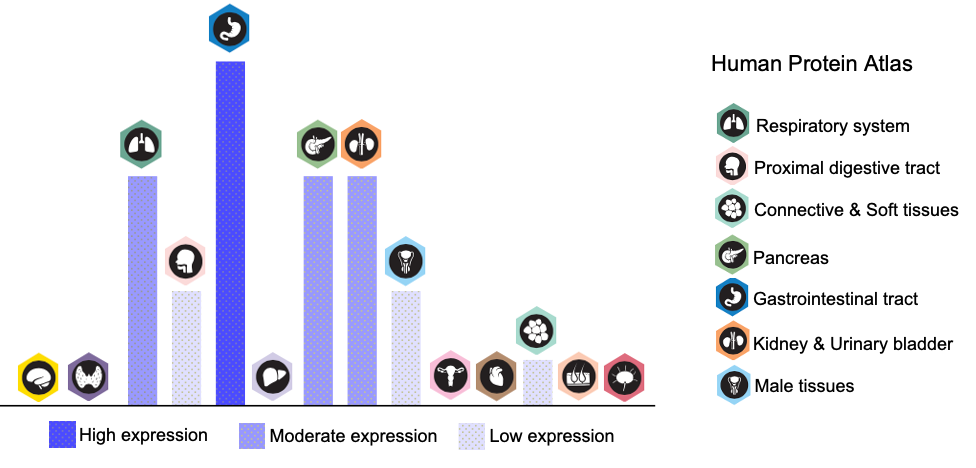
**

**Figure S12. SCTR presents relevant expression in several human healthy organs (respiratory system; gastrointestinal tract; pancreas; kidney and urinary bladder).** According to the Human Protein Atlas, SCTR exhibits high expression in the gastrointestinal tract, moderate expression in the respiratory system, pancreas, kidney, and urinary bladder, and low to vestigial expression in the proximal digestive tract, male tissues, and connective and soft tissues. Expression levels are defined as high, moderate or low, according to the Human Protein Atlas (www.proteinatlas.org/).


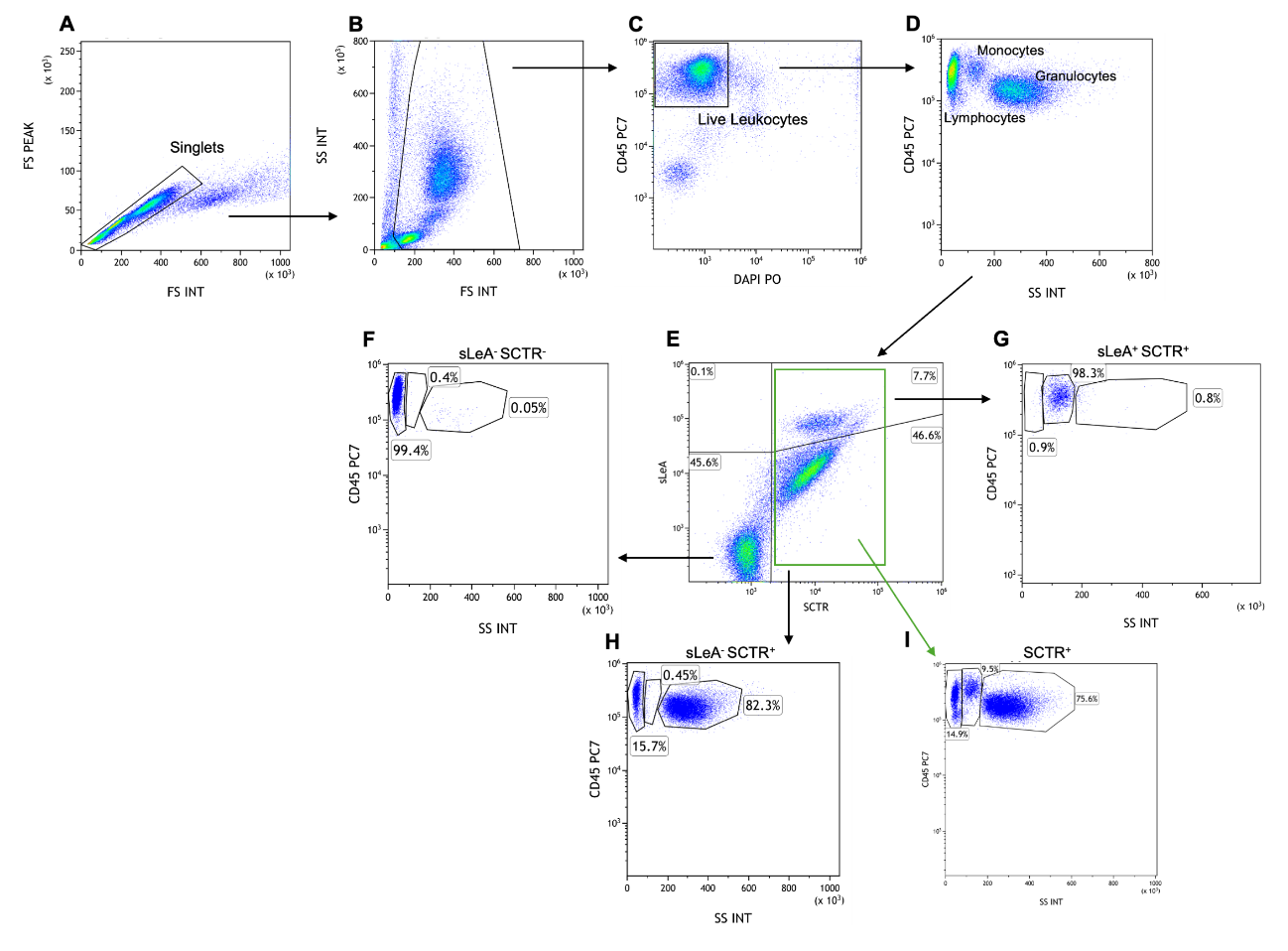


**Figure S13. Flow cytometric gate strategy to identify sLeA and SCTR positive leukocytes in peripheral blood of healthy donors.** **A)** Singlets are selected according to FSC-H versus FSC-A. **B)** Leukocytes are selected, and erythrocytes and debris were excluded using SSC-A versus FSC-A. **C)** Live leukocytes are isolated by selecting the CD45 positive and DAPI negative cells. **D)** Live leukocytes are subtyped into lymphocytes, monocytes, and granulocytes according to CD45 expression and SSC-A dispersion. **E)** sLeA+/SCTR+ leukocytes are isolated using fluorochrome-coupled specific antibodies. **F)** Isolating the double negative leukocytes for sLeA and SCTR, around 100% of the cells are lymphocytes. **G)** Isolating the double positive leukocytes for sLeA and SCTR, 22.5% of the cells are lymphocytes, 67.6% are monocytes, and 3.0% are granulocytes. **H)** Isolating the sLeA negative and SCTR positive leukocytes, 15.7% of the cells are lymphocytes, 0.45% are monocytes, and 82.3% are granulocytes. **I)** Isolating the SCTR positive leukocytes, 17.2% of the cells are lymphocytes, 8.6% are monocytes, and 72.1% are granulocytes.
